# Search for Higgs boson pair production in the $b\bar{b}b\bar{b}$ final state from *pp* collisions at $\sqrt{s} = 8$ TeVwith the ATLAS detector

**DOI:** 10.1140/epjc/s10052-015-3628-x

**Published:** 2015-09-09

**Authors:** G. Aad, B. Abbott, J. Abdallah, O. Abdinov, R. Aben, M. Abolins, O. S. AbouZeid, H. Abramowicz, H. Abreu, R. Abreu, Y. Abulaiti, B. S. Acharya, L. Adamczyk, D. L. Adams, J. Adelman, S. Adomeit, T. Adye, A. A. Affolder, T. Agatonovic-Jovin, J. A. Aguilar-Saavedra, S. P. Ahlen, F. Ahmadov, G. Aielli, H. Akerstedt, T. P. A. Åkesson, G. Akimoto, A. V. Akimov, G. L. Alberghi, J. Albert, S. Albrand, M. J. Alconada Verzini, M. Aleksa, I. N. Aleksandrov, C. Alexa, G. Alexander, T. Alexopoulos, M. Alhroob, G. Alimonti, L. Alio, J. Alison, S. P. Alkire, B. M. M. Allbrooke, P. P. Allport, A. Aloisio, A. Alonso, F. Alonso, C. Alpigiani, A. Altheimer, B. Alvarez Gonzalez, D. Álvarez Piqueras, M. G. Alviggi, B. T. Amadio, K. Amako, Y. Amaral Coutinho, C. Amelung, D. Amidei, S. P. Amor Dos Santos, A. Amorim, S. Amoroso, N. Amram, G. Amundsen, C. Anastopoulos, L. S. Ancu, N. Andari, T. Andeen, C. F. Anders, G. Anders, J. K. Anders, K. J. Anderson, A. Andreazza, V. Andrei, S. Angelidakis, I. Angelozzi, P. Anger, A. Angerami, F. Anghinolfi, A. V. Anisenkov, N. Anjos, A. Annovi, M. Antonelli, A. Antonov, J. Antos, F. Anulli, M. Aoki, L. Aperio Bella, G. Arabidze, Y. Arai, J. P. Araque, A. T. H. Arce, F. A. Arduh, J-F. Arguin, S. Argyropoulos, M. Arik, A. J. Armbruster, O. Arnaez, V. Arnal, H. Arnold, M. Arratia, O. Arslan, A. Artamonov, G. Artoni, S. Asai, N. Asbah, A. Ashkenazi, B. Åsman, L. Asquith, K. Assamagan, R. Astalos, M. Atkinson, N. B. Atlay, B. Auerbach, K. Augsten, M. Aurousseau, G. Avolio, B. Axen, M. K. Ayoub, G. Azuelos, M. A. Baak, A. E. Baas, C. Bacci, H. Bachacou, K. Bachas, M. Backes, M. Backhaus, P. Bagiacchi, P. Bagnaia, Y. Bai, T. Bain, J. T. Baines, O. K. Baker, P. Balek, T. Balestri, F. Balli, E. Banas, Sw. Banerjee, A. A. E. Bannoura, H. S. Bansil, L. Barak, E. L. Barberio, D. Barberis, M. Barbero, T. Barillari, M. Barisonzi, T. Barklow, N. Barlow, S. L. Barnes, B. M. Barnett, R. M. Barnett, Z. Barnovska, A. Baroncelli, G. Barone, A. J. Barr, F. Barreiro, J. Barreiro Guimarães da Costa, R. Bartoldus, A. E. Barton, P. Bartos, A. Basalaev, A. Bassalat, A. Basye, R. L. Bates, S. J. Batista, J. R. Batley, M. Battaglia, M. Bauce, F. Bauer, H. S. Bawa, J. B. Beacham, M. D. Beattie, T. Beau, P. H. Beauchemin, R. Beccherle, P. Bechtle, H. P. Beck, K. Becker, M. Becker, S. Becker, M. Beckingham, C. Becot, A. J. Beddall, A. Beddall, V. A. Bednyakov, C. P. Bee, L. J. Beemster, T. A. Beermann, M. Begel, J. K. Behr, C. Belanger-Champagne, W. H. Bell, G. Bella, L. Bellagamba, A. Bellerive, M. Bellomo, K. Belotskiy, O. Beltramello, O. Benary, D. Benchekroun, M. Bender, K. Bendtz, N. Benekos, Y. Benhammou, E. Benhar Noccioli, J. A. Benitez Garcia, D. P. Benjamin, J. R. Bensinger, S. Bentvelsen, L. Beresford, M. Beretta, D. Berge, E. Bergeaas Kuutmann, N. Berger, F. Berghaus, J. Beringer, C. Bernard, N. R. Bernard, C. Bernius, F. U. Bernlochner, T. Berry, P. Berta, C. Bertella, G. Bertoli, F. Bertolucci, C. Bertsche, D. Bertsche, M. I. Besana, G. J. Besjes, O. Bessidskaia Bylund, M. Bessner, N. Besson, C. Betancourt, S. Bethke, A. J. Bevan, W. Bhimji, R. M. Bianchi, L. Bianchini, M. Bianco, O. Biebel, D. Biedermann, S. P. Bieniek, M. Biglietti, J. Bilbao De Mendizabal, H. Bilokon, M. Bindi, S. Binet, A. Bingul, C. Bini, C. W. Black, J. E. Black, K. M. Black, D. Blackburn, R. E. Blair, J.-B. Blanchard, J. E. Blanco, T. Blazek, I. Bloch, C. Blocker, W. Blum, U. Blumenschein, G. J. Bobbink, V. S. Bobrovnikov, S. S. Bocchetta, A. Bocci, C. Bock, M. Boehler, J. A. Bogaerts, D. Bogavac, A. G. Bogdanchikov, C. Bohm, V. Boisvert, T. Bold, V. Boldea, A. S. Boldyrev, M. Bomben, M. Bona, M. Boonekamp, A. Borisov, G. Borissov, S. Borroni, J. Bortfeldt, V. Bortolotto, K. Bos, D. Boscherini, M. Bosman, J. Boudreau, J. Bouffard, E. V. Bouhova-Thacker, D. Boumediene, C. Bourdarios, N. Bousson, A. Boveia, J. Boyd, I. R. Boyko, I. Bozic, J. Bracinik, A. Brandt, G. Brandt, O. Brandt, U. Bratzler, B. Brau, J. E. Brau, H. M. Braun, S. F. Brazzale, W. D. Breaden Madden, K. Brendlinger, A. J. Brennan, L. Brenner, R. Brenner, S. Bressler, K. Bristow, T. M. Bristow, D. Britton, D. Britzger, F. M. Brochu, I. Brock, R. Brock, J. Bronner, G. Brooijmans, T. Brooks, W. K. Brooks, J. Brosamer, E. Brost, J. Brown, P. A. Bruckman de Renstrom, D. Bruncko, R. Bruneliere, A. Bruni, G. Bruni, M. Bruschi, N. Bruscino, L. Bryngemark, T. Buanes, Q. Buat, P. Buchholz, A. G. Buckley, S. I. Buda, I. A. Budagov, F. Buehrer, L. Bugge, M. K. Bugge, O. Bulekov, D. Bullock, H. Burckhart, S. Burdin, B. Burghgrave, S. Burke, I. Burmeister, E. Busato, D. Büscher, V. Büscher, P. Bussey, J. M. Butler, A. I. Butt, C. M. Buttar, J. M. Butterworth, P. Butti, W. Buttinger, A. Buzatu, A. R. Buzykaev, S. Cabrera Urbán, D. Caforio, V. M. Cairo, O. Cakir, P. Calafiura, A. Calandri, G. Calderini, P. Calfayan, L. P. Caloba, D. Calvet, S. Calvet, R. Camacho Toro, S. Camarda, P. Camarri, D. Cameron, L. M. Caminada, R. Caminal Armadans, S. Campana, M. Campanelli, A. Campoverde, V. Canale, A. Canepa, M. Cano Bret, J. Cantero, R. Cantrill, T. Cao, M. D. M. Capeans Garrido, I. Caprini, M. Caprini, M. Capua, R. Caputo, R. Cardarelli, F. Cardillo, T. Carli, G. Carlino, L. Carminati, S. Caron, E. Carquin, G. D. Carrillo-Montoya, J. R. Carter, J. Carvalho, D. Casadei, M. P. Casado, M. Casolino, E. Castaneda-Miranda, A. Castelli, V. Castillo Gimenez, N. F. Castro, P. Catastini, A. Catinaccio, J. R. Catmore, A. Cattai, J. Caudron, V. Cavaliere, D. Cavalli, M. Cavalli-Sforza, V. Cavasinni, F. Ceradini, B. C. Cerio, K. Cerny, A. S. Cerqueira, A. Cerri, L. Cerrito, F. Cerutti, M. Cerv, A. Cervelli, S. A. Cetin, A. Chafaq, D. Chakraborty, I. Chalupkova, P. Chang, B. Chapleau, J. D. Chapman, D. G. Charlton, C. C. Chau, C. A. Chavez Barajas, S. Cheatham, A. Chegwidden, S. Chekanov, S. V. Chekulaev, G. A. Chelkov, M. A. Chelstowska, C. Chen, H. Chen, K. Chen, L. Chen, S. Chen, X. Chen, Y. Chen, H. C. Cheng, Y. Cheng, A. Cheplakov, E. Cheremushkina, R. Cherkaoui El Moursli, V. Chernyatin, E. Cheu, L. Chevalier, V. Chiarella, J. T. Childers, G. Chiodini, A. S. Chisholm, R. T. Chislett, A. Chitan, M. V. Chizhov, K. Choi, S. Chouridou, B. K. B. Chow, V. Christodoulou, D. Chromek-Burckhart, J. Chudoba, A. J. Chuinard, J. J. Chwastowski, L. Chytka, G. Ciapetti, A. K. Ciftci, D. Cinca, V. Cindro, I. A. Cioara, A. Ciocio, Z. H. Citron, M. Ciubancan, A. Clark, B. L. Clark, P. J. Clark, R. N. Clarke, W. Cleland, C. Clement, Y. Coadou, M. Cobal, A. Coccaro, J. Cochran, L. Coffey, J. G. Cogan, B. Cole, S. Cole, A. P. Colijn, J. Collot, T. Colombo, G. Compostella, P. Conde Muiño, E. Coniavitis, S. H. Connell, I. A. Connelly, S. M. Consonni, V. Consorti, S. Constantinescu, C. Conta, G. Conti, F. Conventi, M. Cooke, B. D. Cooper, A. M. Cooper-Sarkar, T. Cornelissen, M. Corradi, F. Corriveau, A. Corso-Radu, A. Cortes-Gonzalez, G. Cortiana, G. Costa, M. J. Costa, D. Costanzo, D. Côté, G. Cottin, G. Cowan, B. E. Cox, K. Cranmer, G. Cree, S. Crépé-Renaudin, F. Crescioli, W. A. Cribbs, M. Crispin Ortuzar, M. Cristinziani, V. Croft, G. Crosetti, T. Cuhadar Donszelmann, J. Cummings, M. Curatolo, C. Cuthbert, H. Czirr, P. Czodrowski, S. D’Auria, M. D’Onofrio, M. J. Da Cunha Sargedas De Sousa, C. Da Via, W. Dabrowski, A. Dafinca, T. Dai, O. Dale, F. Dallaire, C. Dallapiccola, M. Dam, J. R. Dandoy, N. P. Dang, A. C. Daniells, M. Danninger, M. Dano Hoffmann, V. Dao, G. Darbo, S. Darmora, J. Dassoulas, A. Dattagupta, W. Davey, C. David, T. Davidek, E. Davies, M. Davies, P. Davison, Y. Davygora, E. Dawe, I. Dawson, R. K. Daya-Ishmukhametova, K. De, R. de Asmundis, S. De Castro, S. De Cecco, N. De Groot, P. de Jong, H. De la Torre, F. De Lorenzi, L. De Nooij, D. De Pedis, A. De Salvo, U. De Sanctis, A. De Santo, J. B. De Vivie De Regie, W. J. Dearnaley, R. Debbe, C. Debenedetti, D. V. Dedovich, I. Deigaard, J. Del Peso, T. Del Prete, D. Delgove, F. Deliot, C. M. Delitzsch, M. Deliyergiyev, A. Dell’Acqua, L. Dell’Asta, M. Dell’Orso, M. Della Pietra, D. della Volpe, M. Delmastro, P. A. Delsart, C. Deluca, D. A. DeMarco, S. Demers, M. Demichev, A. Demilly, S. P. Denisov, D. Derendarz, J. E. Derkaoui, F. Derue, P. Dervan, K. Desch, C. Deterre, P. O. Deviveiros, A. Dewhurst, S. Dhaliwal, A. Di Ciaccio, L. Di Ciaccio, A. Di Domenico, C. Di Donato, A. Di Girolamo, B. Di Girolamo, A. Di Mattia, B. Di Micco, R. Di Nardo, A. Di Simone, R. Di Sipio, D. Di Valentino, C. Diaconu, M. Diamond, F. A. Dias, M. A. Diaz, E. B. Diehl, J. Dietrich, S. Diglio, A. Dimitrievska, J. Dingfelder, P. Dita, S. Dita, F. Dittus, F. Djama, T. Djobava, J. I. Djuvsland, M. A. B. do Vale, D. Dobos, M. Dobre, C. Doglioni, T. Dohmae, J. Dolejsi, Z. Dolezal, B. A. Dolgoshein, M. Donadelli, S. Donati, P. Dondero, J. Donini, J. Dopke, A. Doria, M. T. Dova, A. T. Doyle, E. Drechsler, M. Dris, E. Dubreuil, E. Duchovni, G. Duckeck, O. A. Ducu, D. Duda, A. Dudarev, L. Duflot, L. Duguid, M. Dührssen, M. Dunford, H. Duran Yildiz, M. Düren, A. Durglishvili, D. Duschinger, M. Dyndal, C. Eckardt, K. M. Ecker, R. C. Edgar, W. Edson, N. C. Edwards, W. Ehrenfeld, T. Eifert, G. Eigen, K. Einsweiler, T. Ekelof, M. El Kacimi, M. Ellert, S. Elles, F. Ellinghaus, A. A. Elliot, N. Ellis, J. Elmsheuser, M. Elsing, D. Emeliyanov, Y. Enari, O. C. Endner, M. Endo, J. Erdmann, A. Ereditato, G. Ernis, J. Ernst, M. Ernst, S. Errede, E. Ertel, M. Escalier, H. Esch, C. Escobar, B. Esposito, A. I. Etienvre, E. Etzion, H. Evans, A. Ezhilov, L. Fabbri, G. Facini, R. M. Fakhrutdinov, S. Falciano, R. J. Falla, J. Faltova, Y. Fang, M. Fanti, A. Farbin, A. Farilla, T. Farooque, S. Farrell, S. M. Farrington, P. Farthouat, F. Fassi, P. Fassnacht, D. Fassouliotis, M. Faucci Giannelli, A. Favareto, L. Fayard, P. Federic, O. L. Fedin, W. Fedorko, S. Feigl, L. Feligioni, C. Feng, E. J. Feng, H. Feng, A. B. Fenyuk, L. Feremenga, P. Fernandez Martinez, S. Fernandez Perez, J. Ferrando, A. Ferrari, P. Ferrari, R. Ferrari, D. E. Ferreira de Lima, A. Ferrer, D. Ferrere, C. Ferretti, A. Ferretto Parodi, M. Fiascaris, F. Fiedler, A. Filipčič, M. Filipuzzi, F. Filthaut, M. Fincke-Keeler, K. D. Finelli, M. C. N. Fiolhais, L. Fiorini, A. Firan, A. Fischer, C. Fischer, J. Fischer, W. C. Fisher, E. A. Fitzgerald, I. Fleck, P. Fleischmann, S. Fleischmann, G. T. Fletcher, G. Fletcher, R. R. M. Fletcher, T. Flick, A. Floderus, L. R. Flores Castillo, M. J. Flowerdew, A. Formica, A. Forti, D. Fournier, H. Fox, S. Fracchia, P. Francavilla, M. Franchini, D. Francis, L. Franconi, M. Franklin, M. Frate, M. Fraternali, D. Freeborn, S. T. French, F. Friedrich, D. Froidevaux, J. A. Frost, C. Fukunaga, E. Fullana Torregrosa, B. G. Fulsom, J. Fuster, C. Gabaldon, O. Gabizon, A. Gabrielli, A. Gabrielli, S. Gadatsch, S. Gadomski, G. Gagliardi, P. Gagnon, C. Galea, B. Galhardo, E. J. Gallas, B. J. Gallop, P. Gallus, G. Galster, K. K. Gan, J. Gao, Y. Gao, Y. S. Gao, F. M. Garay Walls, F. Garberson, C. García, J. E. García Navarro, M. Garcia-Sciveres, R. W. Gardner, N. Garelli, V. Garonne, C. Gatti, A. Gaudiello, G. Gaudio, B. Gaur, L. Gauthier, P. Gauzzi, I. L. Gavrilenko, C. Gay, G. Gaycken, E. N. Gazis, P. Ge, Z. Gecse, C. N. P. Gee, D. A. A. Geerts, Ch. Geich-Gimbel, M. P. Geisler, C. Gemme, M. H. Genest, S. Gentile, M. George, S. George, D. Gerbaudo, A. Gershon, H. Ghazlane, B. Giacobbe, S. Giagu, V. Giangiobbe, P. Giannetti, B. Gibbard, S. M. Gibson, M. Gilchriese, T. P. S. Gillam, D. Gillberg, G. Gilles, D. M. Gingrich, N. Giokaris, M. P. Giordani, F. M. Giorgi, F. M. Giorgi, P. F. Giraud, P. Giromini, D. Giugni, C. Giuliani, M. Giulini, B. K. Gjelsten, S. Gkaitatzis, I. Gkialas, E. L. Gkougkousis, L. K. Gladilin, C. Glasman, J. Glatzer, P. C. F. Glaysher, A. Glazov, M. Goblirsch-Kolb, J. R. Goddard, J. Godlewski, S. Goldfarb, T. Golling, D. Golubkov, A. Gomes, R. Gonçalo, J. Goncalves Pinto Firmino Da Costa, L. Gonella, S. González de la Hoz, G. Gonzalez Parra, S. Gonzalez-Sevilla, L. Goossens, P. A. Gorbounov, H. A. Gordon, I. Gorelov, B. Gorini, E. Gorini, A. Gorišek, E. Gornicki, A. T. Goshaw, C. Gössling, M. I. Gostkin, D. Goujdami, A. G. Goussiou, N. Govender, E. Gozani, H. M. X. Grabas, L. Graber, I. Grabowska-Bold, P. Grafström, K-J. Grahn, J. Gramling, E. Gramstad, S. Grancagnolo, V. Grassi, V. Gratchev, H. M. Gray, E. Graziani, Z. D. Greenwood, K. Gregersen, I. M. Gregor, P. Grenier, J. Griffiths, A. A. Grillo, K. Grimm, S. Grinstein, Ph. Gris, J.-F. Grivaz, J. P. Grohs, A. Grohsjean, E. Gross, J. Grosse-Knetter, G. C. Grossi, Z. J. Grout, L. Guan, J. Guenther, F. Guescini, D. Guest, O. Gueta, E. Guido, T. Guillemin, S. Guindon, U. Gul, C. Gumpert, J. Guo, S. Gupta, G. Gustavino, P. Gutierrez, N. G. Gutierrez Ortiz, C. Gutschow, C. Guyot, C. Gwenlan, C. B. Gwilliam, A. Haas, C. Haber, H. K. Hadavand, N. Haddad, P. Haefner, S. Hageböck, Z. Hajduk, H. Hakobyan, M. Haleem, J. Haley, D. Hall, G. Halladjian, G. D. Hallewell, K. Hamacher, P. Hamal, K. Hamano, M. Hamer, A. Hamilton, G. N. Hamity, P. G. Hamnett, L. Han, K. Hanagaki, K. Hanawa, M. Hance, P. Hanke, R. Hanna, J. B. Hansen, J. D. Hansen, M. C. Hansen, P. H. Hansen, K. Hara, A. S. Hard, T. Harenberg, F. Hariri, S. Harkusha, R. D. Harrington, P. F. Harrison, F. Hartjes, M. Hasegawa, S. Hasegawa, Y. Hasegawa, A. Hasib, S. Hassani, S. Haug, R. Hauser, L. Hauswald, M. Havranek, C. M. Hawkes, R. J. Hawkings, A. D. Hawkins, T. Hayashi, D. Hayden, C. P. Hays, J. M. Hays, H. S. Hayward, S. J. Haywood, S. J. Head, T. Heck, V. Hedberg, L. Heelan, S. Heim, T. Heim, B. Heinemann, L. Heinrich, J. Hejbal, L. Helary, S. Hellman, D. Hellmich, C. Helsens, J. Henderson, R. C. W. Henderson, Y. Heng, C. Hengler, A. Henrichs, A. M. Henriques Correia, S. Henrot-Versille, G. H. Herbert, Y. Hernández Jiménez, R. Herrberg-Schubert, G. Herten, R. Hertenberger, L. Hervas, G. G. Hesketh, N. P. Hessey, J. W. Hetherly, R. Hickling, E. Higón-Rodriguez, E. Hill, J. C. Hill, K. H. Hiller, S. J. Hillier, I. Hinchliffe, E. Hines, R. R. Hinman, M. Hirose, D. Hirschbuehl, J. Hobbs, N. Hod, M. C. Hodgkinson, P. Hodgson, A. Hoecker, M. R. Hoeferkamp, F. Hoenig, M. Hohlfeld, D. Hohn, T. R. Holmes, M. Homann, T. M. Hong, L. Hooft van Huysduynen, W. H. Hopkins, Y. Horii, A. J. Horton, J-Y. Hostachy, S. Hou, A. Hoummada, J. Howard, J. Howarth, M. Hrabovsky, I. Hristova, J. Hrivnac, T. Hryn’ova, A. Hrynevich, C. Hsu, P. J. Hsu, S.-C. Hsu, D. Hu, Q. Hu, X. Hu, Y. Huang, Z. Hubacek, F. Hubaut, F. Huegging, T. B. Huffman, E. W. Hughes, G. Hughes, M. Huhtinen, T. A. Hülsing, N. Huseynov, J. Huston, J. Huth, G. Iacobucci, G. Iakovidis, I. Ibragimov, L. Iconomidou-Fayard, E. Ideal, Z. Idrissi, P. Iengo, O. Igonkina, T. Iizawa, Y. Ikegami, K. Ikematsu, M. Ikeno, Y. Ilchenko, D. Iliadis, N. Ilic, Y. Inamaru, T. Ince, G. Introzzi, P. Ioannou, M. Iodice, K. Iordanidou, V. Ippolito, A. Irles Quiles, C. Isaksson, M. Ishino, M. Ishitsuka, R. Ishmukhametov, C. Issever, S. Istin, J. M. Iturbe Ponce, R. Iuppa, J. Ivarsson, W. Iwanski, H. Iwasaki, J. M. Izen, V. Izzo, S. Jabbar, B. Jackson, M. Jackson, P. Jackson, M. R. Jaekel, V. Jain, K. Jakobs, S. Jakobsen, T. Jakoubek, J. Jakubek, D. O. Jamin, D. K. Jana, E. Jansen, R. W. Jansky, J. Janssen, M. Janus, G. Jarlskog, N. Javadov, T. Javůrek, L. Jeanty, J. Jejelava, G.-Y. Jeng, D. Jennens, P. Jenni, J. Jentzsch, C. Jeske, S. Jézéquel, H. Ji, J. Jia, Y. Jiang, S. Jiggins, J. Jimenez Pena, S. Jin, A. Jinaru, O. Jinnouchi, M. D. Joergensen, P. Johansson, K. A. Johns, K. Jon-And, G. Jones, R. W. L. Jones, T. J. Jones, J. Jongmanns, P. M. Jorge, K. D. Joshi, J. Jovicevic, X. Ju, C. A. Jung, P. Jussel, A. Juste Rozas, M. Kaci, A. Kaczmarska, M. Kado, H. Kagan, M. Kagan, S. J. Kahn, E. Kajomovitz, C. W. Kalderon, S. Kama, A. Kamenshchikov, N. Kanaya, M. Kaneda, S. Kaneti, V. A. Kantserov, J. Kanzaki, B. Kaplan, A. Kapliy, D. Kar, K. Karakostas, A. Karamaoun, N. Karastathis, M. J. Kareem, M. Karnevskiy, S. N. Karpov, Z. M. Karpova, K. Karthik, V. Kartvelishvili, A. N. Karyukhin, L. Kashif, R. D. Kass, A. Kastanas, Y. Kataoka, A. Katre, J. Katzy, K. Kawagoe, T. Kawamoto, G. Kawamura, S. Kazama, V. F. Kazanin, M. Y. Kazarinov, R. Keeler, R. Kehoe, J. S. Keller, J. J. Kempster, H. Keoshkerian, O. Kepka, B. P. Kerševan, S. Kersten, R. A. Keyes, F. Khalil-zada, H. Khandanyan, A. Khanov, A. G. Kharlamov, T. J. Khoo, V. Khovanskiy, E. Khramov, J. Khubua, H. Y. Kim, H. Kim, S. H. Kim, Y. Kim, N. Kimura, O. M. Kind, B. T. King, M. King, S. B. King, J. Kirk, A. E. Kiryunin, T. Kishimoto, D. Kisielewska, F. Kiss, K. Kiuchi, O. Kivernyk, E. Kladiva, M. H. Klein, M. Klein, U. Klein, K. Kleinknecht, P. Klimek, A. Klimentov, R. Klingenberg, J. A. Klinger, T. Klioutchnikova, E.-E. Kluge, P. Kluit, S. Kluth, E. Kneringer, E. B. F. G. Knoops, A. Knue, A. Kobayashi, D. Kobayashi, T. Kobayashi, M. Kobel, M. Kocian, P. Kodys, T. Koffas, E. Koffeman, L. A. Kogan, S. Kohlmann, Z. Kohout, T. Kohriki, T. Koi, H. Kolanoski, I. Koletsou, A. A. Komar, Y. Komori, T. Kondo, N. Kondrashova, K. Köneke, A. C. König, S. König, T. Kono, R. Konoplich, N. Konstantinidis, R. Kopeliansky, S. Koperny, L. Köpke, A. K. Kopp, K. Korcyl, K. Kordas, A. Korn, A. A. Korol, I. Korolkov, E. V. Korolkova, O. Kortner, S. Kortner, T. Kosek, V. V. Kostyukhin, V. M. Kotov, A. Kotwal, A. Kourkoumeli-Charalampidi, C. Kourkoumelis, V. Kouskoura, A. Koutsman, R. Kowalewski, T. Z. Kowalski, W. Kozanecki, A. S. Kozhin, V. A. Kramarenko, G. Kramberger, D. Krasnopevtsev, M. W. Krasny, A. Krasznahorkay, J. K. Kraus, A. Kravchenko, S. Kreiss, M. Kretz, J. Kretzschmar, K. Kreutzfeldt, P. Krieger, K. Krizka, K. Kroeninger, H. Kroha, J. Kroll, J. Kroseberg, J. Krstic, U. Kruchonak, H. Krüger, N. Krumnack, Z. V. Krumshteyn, A. Kruse, M. C. Kruse, M. Kruskal, T. Kubota, H. Kucuk, S. Kuday, S. Kuehn, A. Kugel, F. Kuger, A. Kuhl, T. Kuhl, V. Kukhtin, Y. Kulchitsky, S. Kuleshov, M. Kuna, T. Kunigo, A. Kupco, H. Kurashige, Y. A. Kurochkin, R. Kurumida, V. Kus, E. S. Kuwertz, M. Kuze, J. Kvita, T. Kwan, D. Kyriazopoulos, A. La Rosa, J. L. La Rosa Navarro, L. La Rotonda, C. Lacasta, F. Lacava, J. Lacey, H. Lacker, D. Lacour, V. R. Lacuesta, E. Ladygin, R. Lafaye, B. Laforge, T. Lagouri, S. Lai, L. Lambourne, S. Lammers, C. L. Lampen, W. Lampl, E. Lançon, U. Landgraf, M. P. J. Landon, V. S. Lang, J. C. Lange, A. J. Lankford, F. Lanni, K. Lantzsch, A. Lanza, S. Laplace, C. Lapoire, J. F. Laporte, T. Lari, F. Lasagni Manghi, M. Lassnig, P. Laurelli, W. Lavrijsen, A. T. Law, P. Laycock, T. Lazovich, O. Le Dortz, E. Le Guirriec, E. Le Menedeu, M. LeBlanc, T. LeCompte, F. Ledroit-Guillon, C. A. Lee, S. C. Lee, L. Lee, G. Lefebvre, M. Lefebvre, F. Legger, C. Leggett, A. Lehan, G. Lehmann Miotto, X. Lei, W. A. Leight, A. Leisos, A. G. Leister, M. A. L. Leite, R. Leitner, D. Lellouch, B. Lemmer, K. J. C. Leney, T. Lenz, B. Lenzi, R. Leone, S. Leone, C. Leonidopoulos, S. Leontsinis, C. Leroy, C. G. Lester, M. Levchenko, J. Levêque, D. Levin, L. J. Levinson, M. Levy, A. Lewis, A. M. Leyko, M. Leyton, B. Li, H. Li, H. L. Li, L. Li, L. Li, S. Li, Y. Li, Z. Liang, H. Liao, B. Liberti, A. Liblong, P. Lichard, K. Lie, J. Liebal, W. Liebig, C. Limbach, A. Limosani, S. C. Lin, T. H. Lin, F. Linde, B. E. Lindquist, J. T. Linnemann, E. Lipeles, A. Lipniacka, M. Lisovyi, T. M. Liss, D. Lissauer, A. Lister, A. M. Litke, B. Liu, D. Liu, H. Liu, J. Liu, J. B. Liu, K. Liu, L. Liu, M. Liu, M. Liu, Y. Liu, M. Livan, A. Lleres, J. Llorente Merino, S. L. Lloyd, F. Lo Sterzo, E. Lobodzinska, P. Loch, W. S. Lockman, F. K. Loebinger, A. E. Loevschall-Jensen, A. Loginov, T. Lohse, K. Lohwasser, M. Lokajicek, B. A. Long, J. D. Long, R. E. Long, K. A. Looper, L. Lopes, D. Lopez Mateos, B. Lopez Paredes, I. Lopez Paz, J. Lorenz, N. Lorenzo Martinez, M. Losada, P. Loscutoff, P. J. Lösel, X. Lou, A. Lounis, J. Love, P. A. Love, N. Lu, H. J. Lubatti, C. Luci, A. Lucotte, F. Luehring, W. Lukas, L. Luminari, O. Lundberg, B. Lund-Jensen, D. Lynn, R. Lysak, E. Lytken, H. Ma, L. L. Ma, G. Maccarrone, A. Macchiolo, C. M. Macdonald, J. Machado Miguens, D. Macina, D. Madaffari, R. Madar, H. J. Maddocks, W. F. Mader, A. Madsen, S. Maeland, T. Maeno, A. Maevskiy, E. Magradze, K. Mahboubi, J. Mahlstedt, C. Maiani, C. Maidantchik, A. A. Maier, T. Maier, A. Maio, S. Majewski, Y. Makida, N. Makovec, B. Malaescu, Pa. Malecki, V. P. Maleev, F. Malek, U. Mallik, D. Malon, C. Malone, S. Maltezos, V. M. Malyshev, S. Malyukov, J. Mamuzic, G. Mancini, B. Mandelli, L. Mandelli, I. Mandić, R. Mandrysch, J. Maneira, A. Manfredini, L. Manhaes de Andrade Filho, J. Manjarres Ramos, A. Mann, P. M. Manning, A. Manousakis-Katsikakis, B. Mansoulie, R. Mantifel, M. Mantoani, L. Mapelli, L. March, G. Marchiori, M. Marcisovsky, C. P. Marino, M. Marjanovic, D. E. Marley, F. Marroquim, S. P. Marsden, Z. Marshall, L. F. Marti, S. Marti-Garcia, B. Martin, T. A. Martin, V. J. Martin, B. Martin dit Latour, M. Martinez, S. Martin-Haugh, V. S. Martoiu, A. C. Martyniuk, M. Marx, F. Marzano, A. Marzin, L. Masetti, T. Mashimo, R. Mashinistov, J. Masik, A. L. Maslennikov, I. Massa, L. Massa, N. Massol, P. Mastrandrea, A. Mastroberardino, T. Masubuchi, P. Mättig, J. Mattmann, J. Maurer, S. J. Maxfield, D. A. Maximov, R. Mazini, S. M. Mazza, L. Mazzaferro, G. Mc Goldrick, S. P. Mc Kee, A. McCarn, R. L. McCarthy, T. G. McCarthy, N. A. McCubbin, K. W. McFarlane, J. A. Mcfayden, G. Mchedlidze, S. J. McMahon, R. A. McPherson, M. Medinnis, S. Meehan, S. Mehlhase, A. Mehta, K. Meier, C. Meineck, B. Meirose, B. R. Mellado Garcia, F. Meloni, A. Mengarelli, S. Menke, E. Meoni, K. M. Mercurio, S. Mergelmeyer, P. Mermod, L. Merola, C. Meroni, F. S. Merritt, A. Messina, J. Metcalfe, A. S. Mete, C. Meyer, C. Meyer, J-P. Meyer, J. Meyer, R. P. Middleton, S. Miglioranzi, L. Mijović, G. Mikenberg, M. Mikestikova, M. Mikuž, M. Milesi, A. Milic, D. W. Miller, C. Mills, A. Milov, D. A. Milstead, A. A. Minaenko, Y. Minami, I. A. Minashvili, A. I. Mincer, B. Mindur, M. Mineev, Y. Ming, L. M. Mir, T. Mitani, J. Mitrevski, V. A. Mitsou, A. Miucci, P. S. Miyagawa, J. U. Mjörnmark, T. Moa, K. Mochizuki, S. Mohapatra, W. Mohr, S. Molander, R. Moles-Valls, K. Mönig, C. Monini, J. Monk, E. Monnier, J. Montejo Berlingen, F. Monticelli, S. Monzani, R. W. Moore, N. Morange, D. Moreno, M. Moreno Llácer, P. Morettini, M. Morgenstern, M. Morii, M. Morinaga, V. Morisbak, S. Moritz, A. K. Morley, G. Mornacchi, J. D. Morris, S. S. Mortensen, A. Morton, L. Morvaj, M. Mosidze, J. Moss, K. Motohashi, R. Mount, E. Mountricha, S. V. Mouraviev, E. J. W. Moyse, S. Muanza, R. D. Mudd, F. Mueller, J. Mueller, K. Mueller, R. S. P. Mueller, T. Mueller, D. Muenstermann, P. Mullen, G. A. Mullier, Y. Munwes, J. A. Murillo Quijada, W. J. Murray, H. Musheghyan, E. Musto, A. G. Myagkov, M. Myska, O. Nackenhorst, J. Nadal, K. Nagai, R. Nagai, Y. Nagai, K. Nagano, A. Nagarkar, Y. Nagasaka, K. Nagata, M. Nagel, E. Nagy, A. M. Nairz, Y. Nakahama, K. Nakamura, T. Nakamura, I. Nakano, H. Namasivayam, R. F. Naranjo Garcia, R. Narayan, T. Naumann, G. Navarro, R. Nayyar, H. A. Neal, P. Yu. Nechaeva, T. J. Neep, P. D. Nef, A. Negri, M. Negrini, S. Nektarijevic, C. Nellist, A. Nelson, S. Nemecek, P. Nemethy, A. A. Nepomuceno, M. Nessi, M. S. Neubauer, M. Neumann, R. M. Neves, P. Nevski, P. R. Newman, D. H. Nguyen, R. B. Nickerson, R. Nicolaidou, B. Nicquevert, J. Nielsen, N. Nikiforou, A. Nikiforov, V. Nikolaenko, I. Nikolic-Audit, K. Nikolopoulos, J. K. Nilsen, P. Nilsson, Y. Ninomiya, A. Nisati, R. Nisius, T. Nobe, M. Nomachi, I. Nomidis, T. Nooney, S. Norberg, M. Nordberg, O. Novgorodova, S. Nowak, M. Nozaki, L. Nozka, K. Ntekas, G. Nunes Hanninger, T. Nunnemann, E. Nurse, F. Nuti, B. J. O’Brien, F. O’grady, D. C. O’Neil, V. O’Shea, F. G. Oakham, H. Oberlack, T. Obermann, J. Ocariz, A. Ochi, I. Ochoa, J. P. Ochoa-Ricoux, S. Oda, S. Odaka, H. Ogren, A. Oh, S. H. Oh, C. C. Ohm, H. Ohman, H. Oide, W. Okamura, H. Okawa, Y. Okumura, T. Okuyama, A. Olariu, S. A. Olivares Pino, D. Oliveira Damazio, E. Oliver Garcia, A. Olszewski, J. Olszowska, A. Onofre, P. U. E. Onyisi, C. J. Oram, M. J. Oreglia, Y. Oren, D. Orestano, N. Orlando, C. Oropeza Barrera, R. S. Orr, B. Osculati, R. Ospanov, G. Otero y Garzon, H. Otono, M. Ouchrif, E. A. Ouellette, F. Ould-Saada, A. Ouraou, K. P. Oussoren, Q. Ouyang, A. Ovcharova, M. Owen, R. E. Owen, V. E. Ozcan, N. Ozturk, K. Pachal, A. Pacheco Pages, C. Padilla Aranda, M. Pagáčová, S. Pagan Griso, E. Paganis, C. Pahl, F. Paige, P. Pais, K. Pajchel, G. Palacino, S. Palestini, M. Palka, D. Pallin, A. Palma, Y. B. Pan, E. Panagiotopoulou, C. E. Pandini, J. G. Panduro Vazquez, P. Pani, S. Panitkin, D. Pantea, L. Paolozzi, Th. D. Papadopoulou, K. Papageorgiou, A. Paramonov, D. Paredes Hernandez, M. A. Parker, K. A. Parker, F. Parodi, J. A. Parsons, U. Parzefall, E. Pasqualucci, S. Passaggio, F. Pastore, Fr. Pastore, G. Pásztor, S. Pataraia, N. D. Patel, J. R. Pater, T. Pauly, J. Pearce, B. Pearson, L. E. Pedersen, M. Pedersen, S. Pedraza Lopez, R. Pedro, S. V. Peleganchuk, D. Pelikan, H. Peng, B. Penning, J. Penwell, D. V. Perepelitsa, E. Perez Codina, M. T. Pérez García-Estañ, L. Perini, H. Pernegger, S. Perrella, R. Peschke, V. D. Peshekhonov, K. Peters, R. F. Y. Peters, B. A. Petersen, T. C. Petersen, E. Petit, A. Petridis, C. Petridou, E. Petrolo, F. Petrucci, N. E. Pettersson, R. Pezoa, P. W. Phillips, G. Piacquadio, E. Pianori, A. Picazio, E. Piccaro, M. Piccinini, M. A. Pickering, R. Piegaia, D. T. Pignotti, J. E. Pilcher, A. D. Pilkington, J. Pina, M. Pinamonti, J. L. Pinfold, A. Pingel, B. Pinto, S. Pires, H. Pirumov, M. Pitt, C. Pizio, L. Plazak, M.-A. Pleier, V. Pleskot, E. Plotnikova, P. Plucinski, D. Pluth, R. Poettgen, L. Poggioli, D. Pohl, G. Polesello, A. Poley, A. Policicchio, R. Polifka, A. Polini, C. S. Pollard, V. Polychronakos, K. Pommès, L. Pontecorvo, B. G. Pope, G. A. Popeneciu, D. S. Popovic, A. Poppleton, S. Pospisil, K. Potamianos, I. N. Potrap, C. J. Potter, C. T. Potter, G. Poulard, J. Poveda, V. Pozdnyakov, P. Pralavorio, A. Pranko, S. Prasad, S. Prell, D. Price, L. E. Price, M. Primavera, S. Prince, M. Proissl, K. Prokofiev, F. Prokoshin, E. Protopapadaki, S. Protopopescu, J. Proudfoot, M. Przybycien, E. Ptacek, D. Puddu, E. Pueschel, D. Puldon, M. Purohit, P. Puzo, J. Qian, G. Qin, Y. Qin, A. Quadt, D. R. Quarrie, W. B. Quayle, M. Queitsch-Maitland, D. Quilty, S. Raddum, V. Radeka, V. Radescu, S. K. Radhakrishnan, P. Radloff, P. Rados, F. Ragusa, G. Rahal, S. Rajagopalan, M. Rammensee, C. Rangel-Smith, F. Rauscher, S. Rave, T. Ravenscroft, M. Raymond, A. L. Read, N. P. Readioff, D. M. Rebuzzi, A. Redelbach, G. Redlinger, R. Reece, K. Reeves, L. Rehnisch, H. Reisin, M. Relich, C. Rembser, H. Ren, A. Renaud, M. Rescigno, S. Resconi, O. L. Rezanova, P. Reznicek, R. Rezvani, R. Richter, S. Richter, E. Richter-Was, O. Ricken, M. Ridel, P. Rieck, C. J. Riegel, J. Rieger, M. Rijssenbeek, A. Rimoldi, L. Rinaldi, B. Ristić, E. Ritsch, I. Riu, F. Rizatdinova, E. Rizvi, S. H. Robertson, A. Robichaud-Veronneau, D. Robinson, J. E. M. Robinson, A. Robson, C. Roda, S. Roe, O. Røhne, S. Rolli, A. Romaniouk, M. Romano, S. M. Romano Saez, E. Romero Adam, N. Rompotis, M. Ronzani, L. Roos, E. Ros, S. Rosati, K. Rosbach, P. Rose, P. L. Rosendahl, O. Rosenthal, V. Rossetti, E. Rossi, L. P. Rossi, R. Rosten, M. Rotaru, I. Roth, J. Rothberg, D. Rousseau, C. R. Royon, A. Rozanov, Y. Rozen, X. Ruan, F. Rubbo, I. Rubinskiy, V. I. Rud, C. Rudolph, M. S. Rudolph, F. Rühr, A. Ruiz-Martinez, Z. Rurikova, N. A. Rusakovich, A. Ruschke, H. L. Russell, J. P. Rutherfoord, N. Ruthmann, Y. F. Ryabov, M. Rybar, G. Rybkin, N. C. Ryder, A. F. Saavedra, G. Sabato, S. Sacerdoti, A. Saddique, H. F-W. Sadrozinski, R. Sadykov, F. Safai Tehrani, M. Saimpert, H. Sakamoto, Y. Sakurai, G. Salamanna, A. Salamon, M. Saleem, D. Salek, P. H. Sales De Bruin, D. Salihagic, A. Salnikov, J. Salt, D. Salvatore, F. Salvatore, A. Salvucci, A. Salzburger, D. Sampsonidis, A. Sanchez, J. Sánchez, V. Sanchez Martinez, H. Sandaker, R. L. Sandbach, H. G. Sander, M. P. Sanders, M. Sandhoff, C. Sandoval, R. Sandstroem, D. P. C. Sankey, M. Sannino, A. Sansoni, C. Santoni, R. Santonico, H. Santos, I. Santoyo Castillo, K. Sapp, A. Sapronov, J. G. Saraiva, B. Sarrazin, O. Sasaki, Y. Sasaki, K. Sato, G. Sauvage, E. Sauvan, G. Savage, P. Savard, C. Sawyer, L. Sawyer, J. Saxon, C. Sbarra, A. Sbrizzi, T. Scanlon, D. A. Scannicchio, M. Scarcella, V. Scarfone, J. Schaarschmidt, P. Schacht, D. Schaefer, R. Schaefer, J. Schaeffer, S. Schaepe, S. Schaetzel, U. Schäfer, A. C. Schaffer, D. Schaile, R. D. Schamberger, V. Scharf, V. A. Schegelsky, D. Scheirich, M. Schernau, C. Schiavi, C. Schillo, M. Schioppa, S. Schlenker, E. Schmidt, K. Schmieden, C. Schmitt, S. Schmitt, S. Schmitt, B. Schneider, Y. J. Schnellbach, U. Schnoor, L. Schoeffel, A. Schoening, B. D. Schoenrock, E. Schopf, A. L. S. Schorlemmer, M. Schott, D. Schouten, J. Schovancova, S. Schramm, M. Schreyer, C. Schroeder, N. Schuh, M. J. Schultens, H.-C. Schultz-Coulon, H. Schulz, M. Schumacher, B. A. Schumm, Ph. Schune, C. Schwanenberger, A. Schwartzman, T. A. Schwarz, Ph. Schwegler, Ph. Schwemling, R. Schwienhorst, J. Schwindling, T. Schwindt, F. G. Sciacca, E. Scifo, G. Sciolla, F. Scuri, F. Scutti, J. Searcy, G. Sedov, E. Sedykh, P. Seema, S. C. Seidel, A. Seiden, F. Seifert, J. M. Seixas, G. Sekhniaidze, K. Sekhon, S. J. Sekula, D. M. Seliverstov, N. Semprini-Cesari, C. Serfon, L. Serin, L. Serkin, T. Serre, M. Sessa, R. Seuster, H. Severini, T. Sfiligoj, F. Sforza, A. Sfyrla, E. Shabalina, M. Shamim, L. Y. Shan, R. Shang, J. T. Shank, M. Shapiro, P. B. Shatalov, K. Shaw, S. M. Shaw, A. Shcherbakova, C. Y. Shehu, P. Sherwood, L. Shi, S. Shimizu, C. O. Shimmin, M. Shimojima, M. Shiyakova, A. Shmeleva, D. Shoaleh Saadi, M. J. Shochet, S. Shojaii, S. Shrestha, E. Shulga, M. A. Shupe, S. Shushkevich, P. Sicho, O. Sidiropoulou, D. Sidorov, A. Sidoti, F. Siegert, Dj. Sijacki, J. Silva, Y. Silver, S. B. Silverstein, V. Simak, O. Simard, Lj. Simic, S. Simion, E. Simioni, B. Simmons, D. Simon, R. Simoniello, P. Sinervo, N. B. Sinev, G. Siragusa, A. N. Sisakyan, S. Yu. Sivoklokov, J. Sjölin, T. B. Sjursen, M. B. Skinner, H. P. Skottowe, P. Skubic, M. Slater, T. Slavicek, M. Slawinska, K. Sliwa, V. Smakhtin, B. H. Smart, L. Smestad, S. Yu. Smirnov, Y. Smirnov, L. N. Smirnova, O. Smirnova, M. N. K. Smith, R. W. Smith, M. Smizanska, K. Smolek, A. A. Snesarev, G. Snidero, S. Snyder, R. Sobie, F. Socher, A. Soffer, D. A. Soh, C. A. Solans, M. Solar, J. Solc, E. Yu. Soldatov, U. Soldevila, A. A. Solodkov, A. Soloshenko, O. V. Solovyanov, V. Solovyev, P. Sommer, H. Y. Song, N. Soni, A. Sood, A. Sopczak, B. Sopko, V. Sopko, V. Sorin, D. Sosa, M. Sosebee, C. L. Sotiropoulou, R. Soualah, A. M. Soukharev, D. South, B. C. Sowden, S. Spagnolo, M. Spalla, F. Spanò, W. R. Spearman, F. Spettel, R. Spighi, G. Spigo, L. A. Spiller, M. Spousta, T. Spreitzer, R. D. St. Denis, S. Staerz, J. Stahlman, R. Stamen, S. Stamm, E. Stanecka, C. Stanescu, M. Stanescu-Bellu, M. M. Stanitzki, S. Stapnes, E. A. Starchenko, J. Stark, P. Staroba, P. Starovoitov, R. Staszewski, P. Stavina, P. Steinberg, B. Stelzer, H. J. Stelzer, O. Stelzer-Chilton, H. Stenzel, S. Stern, G. A. Stewart, J. A. Stillings, M. C. Stockton, M. Stoebe, G. Stoicea, P. Stolte, S. Stonjek, A. R. Stradling, A. Straessner, M. E. Stramaglia, J. Strandberg, S. Strandberg, A. Strandlie, E. Strauss, M. Strauss, P. Strizenec, R. Ströhmer, D. M. Strom, R. Stroynowski, A. Strubig, S. A. Stucci, B. Stugu, N. A. Styles, D. Su, J. Su, R. Subramaniam, A. Succurro, Y. Sugaya, C. Suhr, M. Suk, V. V. Sulin, S. Sultansoy, T. Sumida, S. Sun, X. Sun, J. E. Sundermann, K. Suruliz, G. Susinno, M. R. Sutton, S. Suzuki, Y. Suzuki, M. Svatos, S. Swedish, M. Swiatlowski, I. Sykora, T. Sykora, D. Ta, C. Taccini, K. Tackmann, J. Taenzer, A. Taffard, R. Tafirout, N. Taiblum, H. Takai, R. Takashima, H. Takeda, T. Takeshita, Y. Takubo, M. Talby, A. A. Talyshev, J. Y. C. Tam, K. G. Tan, J. Tanaka, R. Tanaka, S. Tanaka, B. B. Tannenwald, N. Tannoury, S. Tapprogge, S. Tarem, F. Tarrade, G. F. Tartarelli, P. Tas, M. Tasevsky, T. Tashiro, E. Tassi, A. Tavares Delgado, Y. Tayalati, F. E. Taylor, G. N. Taylor, W. Taylor, F. A. Teischinger, M. Teixeira Dias Castanheira, P. Teixeira-Dias, K. K. Temming, H. Ten Kate, P. K. Teng, J. J. Teoh, F. Tepel, S. Terada, K. Terashi, J. Terron, S. Terzo, M. Testa, R. J. Teuscher, J. Therhaag, T. Theveneaux-Pelzer, J. P. Thomas, J. Thomas-Wilsker, E. N. Thompson, P. D. Thompson, R. J. Thompson, A. S. Thompson, L. A. Thomsen, E. Thomson, M. Thomson, R. P. Thun, M. J. Tibbetts, R. E. Ticse Torres, V. O. Tikhomirov, Yu. A. Tikhonov, S. Timoshenko, E. Tiouchichine, P. Tipton, S. Tisserant, T. Todorov, S. Todorova-Nova, J. Tojo, S. Tokár, K. Tokushuku, K. Tollefson, E. Tolley, L. Tomlinson, M. Tomoto, L. Tompkins, K. Toms, E. Torrence, H. Torres, E. Torró Pastor, J. Toth, F. Touchard, D. R. Tovey, T. Trefzger, L. Tremblet, A. Tricoli, I. M. Trigger, S. Trincaz-Duvoid, M. F. Tripiana, W. Trischuk, B. Trocmé, C. Troncon, M. Trottier-McDonald, M. Trovatelli, P. True, L. Truong, M. Trzebinski, A. Trzupek, C. Tsarouchas, J. C-L. Tseng, P. V. Tsiareshka, D. Tsionou, G. Tsipolitis, N. Tsirintanis, S. Tsiskaridze, V. Tsiskaridze, E. G. Tskhadadze, I. I. Tsukerman, V. Tsulaia, S. Tsuno, D. Tsybychev, A. Tudorache, V. Tudorache, A. N. Tuna, S. A. Tupputi, S. Turchikhin, D. Turecek, R. Turra, A. J. Turvey, P. M. Tuts, A. Tykhonov, M. Tylmad, M. Tyndel, I. Ueda, R. Ueno, M. Ughetto, M. Ugland, M. Uhlenbrock, F. Ukegawa, G. Unal, A. Undrus, G. Unel, F. C. Ungaro, Y. Unno, C. Unverdorben, J. Urban, P. Urquijo, P. Urrejola, G. Usai, A. Usanova, L. Vacavant, V. Vacek, B. Vachon, C. Valderanis, N. Valencic, S. Valentinetti, A. Valero, L. Valery, S. Valkar, E. Valladolid Gallego, S. Vallecorsa, J. A. Valls Ferrer, W. Van Den Wollenberg, P. C. Van Der Deijl, R. van der Geer, H. van der Graaf, R. Van Der Leeuw, N. van Eldik, P. van Gemmeren, J. Van Nieuwkoop, I. van Vulpen, M. C. van Woerden, M. Vanadia, W. Vandelli, R. Vanguri, A. Vaniachine, F. Vannucci, G. Vardanyan, R. Vari, E. W. Varnes, T. Varol, D. Varouchas, A. Vartapetian, K. E. Varvell, F. Vazeille, T. Vazquez Schroeder, J. Veatch, L. M. Veloce, F. Veloso, T. Velz, S. Veneziano, A. Ventura, D. Ventura, M. Venturi, N. Venturi, A. Venturini, V. Vercesi, M. Verducci, W. Verkerke, J. C. Vermeulen, A. Vest, M. C. Vetterli, O. Viazlo, I. Vichou, T. Vickey, O. E. Vickey Boeriu, G. H. A. Viehhauser, S. Viel, R. Vigne, M. Villa, M. Villaplana Perez, E. Vilucchi, M. G. Vincter, V. B. Vinogradov, I. Vivarelli, F. Vives Vaque, S. Vlachos, D. Vladoiu, M. Vlasak, M. Vogel, P. Vokac, G. Volpi, M. Volpi, H. von der Schmitt, H. von Radziewski, E. von Toerne, V. Vorobel, K. Vorobev, M. Vos, R. Voss, J. H. Vossebeld, N. Vranjes, M. Vranjes Milosavljevic, V. Vrba, M. Vreeswijk, R. Vuillermet, I. Vukotic, Z. Vykydal, P. Wagner, W. Wagner, H. Wahlberg, S. Wahrmund, J. Wakabayashi, J. Walder, R. Walker, W. Walkowiak, C. Wang, F. Wang, H. Wang, H. Wang, J. Wang, J. Wang, K. Wang, R. Wang, S. M. Wang, T. Wang, X. Wang, C. Wanotayaroj, A. Warburton, C. P. Ward, D. R. Wardrope, M. Warsinsky, A. Washbrook, C. Wasicki, P. M. Watkins, A. T. Watson, I. J. Watson, M. F. Watson, G. Watts, S. Watts, B. M. Waugh, S. Webb, M. S. Weber, S. W. Weber, J. S. Webster, A. R. Weidberg, B. Weinert, J. Weingarten, C. Weiser, H. Weits, P. S. Wells, T. Wenaus, T. Wengler, S. Wenig, N. Wermes, M. Werner, P. Werner, M. Wessels, J. Wetter, K. Whalen, A. M. Wharton, A. White, M. J. White, R. White, S. White, D. Whiteson, F. J. Wickens, W. Wiedenmann, M. Wielers, P. Wienemann, C. Wiglesworth, L. A. M. Wiik-Fuchs, A. Wildauer, H. G. Wilkens, H. H. Williams, S. Williams, C. Willis, S. Willocq, A. Wilson, J. A. Wilson, I. Wingerter-Seez, F. Winklmeier, B. T. Winter, M. Wittgen, J. Wittkowski, S. J. Wollstadt, M. W. Wolter, H. Wolters, B. K. Wosiek, J. Wotschack, M. J. Woudstra, K. W. Wozniak, M. Wu, M. Wu, S. L. Wu, X. Wu, Y. Wu, T. R. Wyatt, B. M. Wynne, S. Xella, D. Xu, L. Xu, B. Yabsley, S. Yacoob, R. Yakabe, M. Yamada, Y. Yamaguchi, A. Yamamoto, S. Yamamoto, T. Yamanaka, K. Yamauchi, Y. Yamazaki, Z. Yan, H. Yang, H. Yang, Y. Yang, W-M. Yao, Y. Yasu, E. Yatsenko, K. H. Yau Wong, J. Ye, S. Ye, I. Yeletskikh, A. L. Yen, E. Yildirim, K. Yorita, R. Yoshida, K. Yoshihara, C. Young, C. J. S. Young, S. Youssef, D. R. Yu, J. Yu, J. M. Yu, J. Yu, L. Yuan, A. Yurkewicz, I. Yusuff, B. Zabinski, R. Zaidan, A. M. Zaitsev, J. Zalieckas, A. Zaman, S. Zambito, L. Zanello, D. Zanzi, C. Zeitnitz, M. Zeman, A. Zemla, K. Zengel, O. Zenin, T. Ženiš, D. Zerwas, D. Zhang, F. Zhang, H. Zhang, J. Zhang, L. Zhang, R. Zhang, X. Zhang, Z. Zhang, X. Zhao, Y. Zhao, Z. Zhao, A. Zhemchugov, J. Zhong, B. Zhou, C. Zhou, L. Zhou, L. Zhou, N. Zhou, C. G. Zhu, H. Zhu, J. Zhu, Y. Zhu, X. Zhuang, K. Zhukov, A. Zibell, D. Zieminska, N. I. Zimine, C. Zimmermann, S. Zimmermann, Z. Zinonos, M. Zinser, M. Ziolkowski, L. Živković, G. Zobernig, A. Zoccoli, M. zur Nedden, G. Zurzolo, L. Zwalinski, ATLAS Collaboration

**Affiliations:** Department of Physics, University of Adelaide, Adelaide, Australia; Physics Department, SUNY Albany, Albany, NY USA; Department of Physics, University of Alberta, Edmonton, AB Canada; Department of Physics, Ankara University, Ankara, Turkey; Istanbul Aydin University, Istanbul, Turkey; Division of Physics, TOBB University of Economics and Technology, Ankara, Turkey; LAPP, CNRS/IN2P3 and Université Savoie Mont Blanc, Annecy-le-Vieux, France; High Energy Physics Division, Argonne National Laboratory, Argonne, IL USA; Department of Physics, University of Arizona, Tucson, AZ USA; Department of Physics, The University of Texas at Arlington, Arlington, TX USA; Physics Department, University of Athens, Athens, Greece; Physics Department, National Technical University of Athens, Zografou, Greece; Institute of Physics, Azerbaijan Academy of Sciences, Baku, Azerbaijan; Institut de Física d’Altes Energies and Departament de Física de la Universitat Autònoma de Barcelona, Barcelona, Spain; Institute of Physics, University of Belgrade, Belgrade, Serbia; Department for Physics and Technology, University of Bergen, Bergen, Norway; Physics Division, Lawrence Berkeley National Laboratory and University of California, Berkeley, CA USA; Department of Physics, Humboldt University, Berlin, Germany; Albert Einstein Center for Fundamental Physics and Laboratory for High Energy Physics, University of Bern, Bern, Switzerland; School of Physics and Astronomy, University of Birmingham, Birmingham, UK; Department of Physics, Bogazici University, Istanbul, Turkey; Department of Physics, Dogus University, Istanbul, Turkey; Department of Physics Engineering, Gaziantep University, Gaziantep, Turkey; INFN Sezione di Bologna, Bologna, Italy; Dipartimento di Fisica e Astronomia, Università di Bologna, Bologna, Italy; Physikalisches Institut, University of Bonn, Bonn, Germany; Department of Physics, Boston University, Boston, MA USA; Department of Physics, Brandeis University, Waltham, MA USA; Universidade Federal do Rio De Janeiro COPPE/EE/IF, Rio de Janeiro, Brazil; Electrical Circuits Department, Federal University of Juiz de Fora (UFJF), Juiz de Fora, Brazil; Federal University of Sao Joao del Rei (UFSJ), Sao Joao del Rei, Brazil; Instituto de Fisica, Universidade de Sao Paulo, São Paulo, Brazil; Physics Department, Brookhaven National Laboratory, Upton, NY USA; National Institute of Physics and Nuclear Engineering, Bucharest, Romania; Physics Department, National Institute for Research and Development of Isotopic and Molecular Technologies, Cluj Napoca, Romania; University Politehnica Bucharest, Bucharest, Romania; West University in Timisoara, Timisoara, Romania; Departamento de Física, Universidad de Buenos Aires, Buenos Aires, Argentina; Cavendish Laboratory, University of Cambridge, Cambridge, UK; Department of Physics, Carleton University, Ottawa, ON Canada; CERN, Geneva, Switzerland; Enrico Fermi Institute, University of Chicago, Chicago, IL USA; Departamento de Física, Pontificia Universidad Católica de Chile, Santiago, Chile; Departamento de Física, Universidad Técnica Federico Santa María, Valparaiso, Chile; Institute of High Energy Physics, Chinese Academy of Sciences, Beijing, China; Department of Modern Physics, University of Science and Technology of China, Hefei, Anhui China; Department of Physics, Nanjing University, Nanjing, Jiangsu China; School of Physics, Shandong University, Shandong, China; Shanghai Key Laboratory for Particle Physics and Cosmology, Department of Physics and Astronomy, Shanghai Jiao Tong University, Shanghai, China; Physics Department, Tsinghua University, Beijing, 100084 China; Laboratoire de Physique Corpusculaire, Clermont Université and Université Blaise Pascal and CNRS/IN2P3, Clermont-Ferrand, France; Nevis Laboratory, Columbia University, Irvington, NY USA; Niels Bohr Institute, University of Copenhagen, Copenhagen, Denmark; INFN Gruppo Collegato di Cosenza, Laboratori Nazionali di Frascati, Frascati, Italy; Dipartimento di Fisica, Università della Calabria, Rende, Italy; AGH University of Science and Technology, Faculty of Physics and Applied Computer Science, Kraków, Poland; Marian Smoluchowski Institute of Physics, Jagiellonian University, Kraków, Poland; Institute of Nuclear Physics, Polish Academy of Sciences, Kraków, Poland; Physics Department, Southern Methodist University, Dallas, TX USA; Physics Department, University of Texas at Dallas, Richardson, TX USA; DESY, Hamburg and Zeuthen, Germany; Institut für Experimentelle Physik IV, Technische Universität Dortmund, Dortmund, Germany; Institut für Kern- und Teilchenphysik, Technische Universität Dresden, Dresden, Germany; Department of Physics, Duke University, Durham, NC USA; SUPA-School of Physics and Astronomy, University of Edinburgh, Edinburgh, UK; INFN Laboratori Nazionali di Frascati, Frascati, Italy; Fakultät für Mathematik und Physik, Albert-Ludwigs-Universität, Freiburg, Germany; Section de Physique, Université de Genève, Geneva, Switzerland; INFN Sezione di Genova, Genoa, Italy; Dipartimento di Fisica, Università di Genova, Genoa, Italy; E. Andronikashvili Institute of Physics, Iv. Javakhishvili Tbilisi State University, Tbilisi, Georgia; High Energy Physics Institute, Tbilisi State University, Tbilisi, Georgia; II Physikalisches Institut, Justus-Liebig-Universität Giessen, Giessen, Germany; SUPA-School of Physics and Astronomy, University of Glasgow, Glasgow, UK; II Physikalisches Institut, Georg-August-Universität, Göttingen, Germany; Laboratoire de Physique Subatomique et de Cosmologie, Université Grenoble-Alpes, CNRS/IN2P3, Grenoble, France; Department of Physics, Hampton University, Hampton, VA USA; Laboratory for Particle Physics and Cosmology, Harvard University, Cambridge, MA USA; Kirchhoff-Institut für Physik, Ruprecht-Karls-Universität Heidelberg, Heidelberg, Germany; Physikalisches Institut, Ruprecht-Karls-Universität Heidelberg, Heidelberg, Germany; ZITI Institut für technische Informatik, Ruprecht-Karls-Universität Heidelberg, Mannheim, Germany; Faculty of Applied Information Science, Hiroshima Institute of Technology, Hiroshima, Japan; Department of Physics, The Chinese University of Hong Kong, Shatin, NT Hong Kong; Department of Physics, The University of Hong Kong, Pok Fu Lam, Hong Kong; Department of Physics, The Hong Kong University of Science and Technology, Clear Water Bay, Kowloon, Hong Kong China; Department of Physics, Indiana University, Bloomington, IN USA; Institut für Astro- und Teilchenphysik, Leopold-Franzens-Universität, Innsbruck, Austria; University of Iowa, Iowa City, IA USA; Department of Physics and Astronomy, Iowa State University, Ames, IA USA; Joint Institute for Nuclear Research, JINR Dubna, Dubna, Russia; KEK, High Energy Accelerator Research Organization, Tsukuba, Japan; Graduate School of Science, Kobe University, Kobe, Japan; Faculty of Science, Kyoto University, Kyoto, Japan; Kyoto University of Education, Kyoto, Japan; Department of Physics, Kyushu University, Fukuoka, Japan; Instituto de Física La Plata, Universidad Nacional de La Plata and CONICET, La Plata, Argentina; Physics Department, Lancaster University, Lancaster, UK; INFN Sezione di Lecce, Lecce, Italy; Dipartimento di Matematica e Fisica, Università del Salento, Lecce, Italy; Oliver Lodge Laboratory, University of Liverpool, Liverpool, UK; Department of Physics, Jožef Stefan Institute and University of Ljubljana, Ljubljana, Slovenia; School of Physics and Astronomy, Queen Mary University of London, London, UK; Department of Physics, Royal Holloway University of London, Surrey, UK; Department of Physics and Astronomy, University College London, London, UK; Louisiana Tech University, Ruston, LA USA; Laboratoire de Physique Nucléaire et de Hautes Energies, UPMC and Université Paris-Diderot and CNRS/IN2P3, Paris, France; Fysiska institutionen, Lunds universitet, Lund, Sweden; Departamento de Fisica Teorica C-15, Universidad Autonoma de Madrid, Madrid, Spain; Institut für Physik, Universität Mainz, Mainz, Germany; School of Physics and Astronomy, University of Manchester, Manchester, UK; CPPM, Aix-Marseille Université and CNRS/IN2P3, Marseille, France; Department of Physics, University of Massachusetts, Amherst, MA USA; Department of Physics, McGill University, Montreal, QC Canada; School of Physics, University of Melbourne, Melbourne, VIC Australia; Department of Physics, The University of Michigan, Ann Arbor, MI USA; Department of Physics and Astronomy, Michigan State University, East Lansing, MI USA; INFN Sezione di Milano, Milan, Italy; Dipartimento di Fisica, Università di Milano, Milan, Italy; B.I. Stepanov Institute of Physics, National Academy of Sciences of Belarus, Minsk, Republic of Belarus; National Scientific and Educational Centre for Particle and High Energy Physics, Minsk, Republic of Belarus; Department of Physics, Massachusetts Institute of Technology, Cambridge, MA USA; Group of Particle Physics, University of Montreal, Montreal, QC Canada; P.N. Lebedev Institute of Physics, Academy of Sciences, Moscow, Russia; Institute for Theoretical and Experimental Physics (ITEP), Moscow, Russia; National Research Nuclear University MEPhI, Moscow, Russia; D.V. Skobeltsyn Institute of Nuclear Physics, M.V. Lomonosov Moscow State University, Moscow, Russia; Fakultät für Physik, Ludwig-Maximilians-Universität München, Munich, Germany; Max-Planck-Institut für Physik (Werner-Heisenberg-Institut), Munich, Germany; Nagasaki Institute of Applied Science, Nagasaki, Japan; Graduate School of Science and Kobayashi-Maskawa Institute, Nagoya University, Nagoya, Japan; INFN Sezione di Napoli, Naples, Italy; Dipartimento di Fisica, Università di Napoli, Naples, Italy; Department of Physics and Astronomy, University of New Mexico, Albuquerque, NM USA; Institute for Mathematics, Astrophysics and Particle Physics, Radboud University Nijmegen/Nikhef, Nijmegen, The Netherlands; Nikhef National Institute for Subatomic Physics and University of Amsterdam, Amsterdam, The Netherlands; Department of Physics, Northern Illinois University, De Kalb, IL USA; Budker Institute of Nuclear Physics, SB RAS, Novosibirsk, Russia; Department of Physics, New York University, New York, NY USA; Ohio State University, Columbus, OH USA; Faculty of Science, Okayama University, Okayama, Japan; Homer L. Dodge Department of Physics and Astronomy, University of Oklahoma, Norman, OK USA; Department of Physics, Oklahoma State University, Stillwater, OK USA; Palacký University, RCPTM, Olomouc, Czech Republic; Center for High Energy Physics, University of Oregon, Eugene, OR USA; LAL, Université Paris-Sud and CNRS/IN2P3, Orsay, France; Graduate School of Science, Osaka University, Osaka, Japan; Department of Physics, University of Oslo, Oslo, Norway; Department of Physics, Oxford University, Oxford, UK; INFN Sezione di Pavia, Pavia, Italy; Dipartimento di Fisica, Università di Pavia, Pavia, Italy; Department of Physics, University of Pennsylvania, Philadelphia, PA USA; National Research Centre “Kurchatov Institute” B.P.Konstantinov Petersburg Nuclear Physics Institute, St. Petersburg, Russia; INFN Sezione di Pisa, Pisa, Italy; Dipartimento di Fisica E. Fermi, Università di Pisa, Pisa, Italy; Department of Physics and Astronomy, University of Pittsburgh, Pittsburgh, PA USA; Laboratorio de Instrumentacao e Fisica Experimental de Particulas, LIP, Lisbon, Portugal; Faculdade de Ciências, Universidade de Lisboa, Lisbon, Portugal; Department of Physics, University of Coimbra, Coimbra, Portugal; Centro de Física Nuclear da Universidade de Lisboa, Lisbon, Portugal; Departamento de Fisica, Universidade do Minho, Braga, Portugal; Departamento de Fisica Teorica y del Cosmos and CAFPE, Universidad de Granada, Granada, Spain; Dep Fisica and CEFITEC of Faculdade de Ciencias e Tecnologia, Universidade Nova de Lisboa, Caparica, Portugal; Institute of Physics, Academy of Sciences of the Czech Republic, Prague, Czech Republic; Czech Technical University in Prague, Prague, Czech Republic; Faculty of Mathematics and Physics, Charles University in Prague, Prague, Czech Republic; State Research Center Institute for High Energy Physics, Protvino, Russia; Particle Physics Department, Rutherford Appleton Laboratory, Didcot, UK; INFN Sezione di Roma, Rome, Italy; Dipartimento di Fisica, Sapienza Università di Roma, Rome, Italy; INFN Sezione di Roma Tor Vergata, Rome, Italy; Dipartimento di Fisica, Università di Roma Tor Vergata, Rome, Italy; INFN Sezione di Roma Tre, Rome, Italy; Dipartimento di Matematica e Fisica, Università Roma Tre, Rome, Italy; Faculté des Sciences Ain Chock, Réseau Universitaire de Physique des Hautes Energies-Université Hassan II, Casablanca, Morocco; Centre National de l’Energie des Sciences Techniques Nucleaires, Rabat, Morocco; Faculté des Sciences Semlalia, Université Cadi Ayyad, LPHEA-Marrakech, Marrakech, Morocco; Faculté des Sciences, Université Mohamed Premier and LPTPM, Oujda, Morocco; Faculté des Sciences, Université Mohammed V-Agdal, Rabat, Morocco; DSM/IRFU (Institut de Recherches sur les Lois Fondamentales de l’Univers), CEA Saclay (Commissariat à l’Energie Atomique et aux Energies Alternatives), Gif-sur-Yvette, France; Santa Cruz Institute for Particle Physics, University of California Santa Cruz, Santa Cruz, CA USA; Department of Physics, University of Washington, Seattle, WA USA; Department of Physics and Astronomy, University of Sheffield, Sheffield, UK; Department of Physics, Shinshu University, Nagano, Japan; Fachbereich Physik, Universität Siegen, Siegen, Germany; Department of Physics, Simon Fraser University, Burnaby, BC Canada; SLAC National Accelerator Laboratory, Stanford, CA USA; Faculty of Mathematics, Physics and Informatics, Comenius University, Bratislava, Slovak Republic; Department of Subnuclear Physics, Institute of Experimental Physics of the Slovak Academy of Sciences, Kosice, Slovak Republic; Department of Physics, University of Cape Town, Cape Town, South Africa; Department of Physics, University of Johannesburg, Johannesburg, South Africa; School of Physics, University of the Witwatersrand, Johannesburg, South Africa; Department of Physics, Stockholm University, Stockholm, Sweden; The Oskar Klein Centre, Stockholm, Sweden; Physics Department, Royal Institute of Technology, Stockholm, Sweden; Departments of Physics and Astronomy and Chemistry, Stony Brook University, Stony Brook, NY USA; Department of Physics and Astronomy, University of Sussex, Brighton, UK; School of Physics, University of Sydney, Sydney, Australia; Institute of Physics, Academia Sinica, Taipei, Taiwan; Department of Physics, Technion: Israel Institute of Technology, Haifa, Israel; Raymond and Beverly Sackler School of Physics and Astronomy, Tel Aviv University, Tel Aviv, Israel; Department of Physics, Aristotle University of Thessaloniki, Thessaloníki, Greece; International Center for Elementary Particle Physics and Department of Physics, The University of Tokyo, Tokyo, Japan; Graduate School of Science and Technology, Tokyo Metropolitan University, Tokyo, Japan; Department of Physics, Tokyo Institute of Technology, Tokyo, Japan; Department of Physics, University of Toronto, Toronto, ON Canada; TRIUMF, Vancouver, BC Canada; Department of Physics and Astronomy, York University, Toronto, ON Canada; Faculty of Pure and Applied Sciences, University of Tsukuba, Tsukuba, Japan; Department of Physics and Astronomy, Tufts University, Medford, MA USA; Centro de Investigaciones, Universidad Antonio Narino, Bogotá, Colombia; Department of Physics and Astronomy, University of California Irvine, Irvine, CA USA; INFN Gruppo Collegato di Udine, Sezione di Trieste, Udine, Italy; ICTP, Trieste, Italy; Dipartimento di Chimica Fisica e Ambiente, Università di Udine, Udine, Italy; Department of Physics, University of Illinois, Urbana, IL USA; Department of Physics and Astronomy, University of Uppsala, Uppsala, Sweden; Instituto de Física Corpuscular (IFIC) and Departamento de Física Atómica, Molecular y Nuclear and Departamento de Ingeniería Electrónica and Instituto de Microelectrónica de Barcelona (IMB-CNM), University of Valencia and CSIC, Valencia, Spain; Department of Physics, University of British Columbia, Vancouver, BC Canada; Department of Physics and Astronomy, University of Victoria, Victoria, BC Canada; Department of Physics, University of Warwick, Coventry, UK; Waseda University, Tokyo, Japan; Department of Particle Physics, The Weizmann Institute of Science, Rehovot, Israel; Department of Physics, University of Wisconsin, Madison, WI USA; Fakultät für Physik und Astronomie, Julius-Maximilians-Universität, Würzburg, Germany; Fachbereich C Physik, Bergische Universität Wuppertal, Wuppertal, Germany; Department of Physics, Yale University, New Haven, CT USA; Yerevan Physics Institute, Yerevan, Armenia; Centre de Calcul de l’Institut National de Physique Nucléaire et de Physique des Particules (IN2P3), Villeurbanne, France; CERN, 1211 Geneva 23, Switzerland

## Abstract

A search for Higgs boson pair production $pp \rightarrow hh$ is performed with 19.5 fb$^{-1}$ of proton–proton collision data at $\sqrt{s}=8$ TeV, which were recorded by the ATLAS detector at the Large Hadron Collider in 2012. The decay products of each Higgs boson are reconstructed as a high-momentum $b\bar{b}$ system with either a pair of small-radius jets or a single large-radius jet, the latter exploiting jet substructure techniques and associated *b*-tagged track-jets. No evidence for resonant or non-resonant Higgs boson pair production is observed. The data are interpreted in the context of the Randall–Sundrum model with a warped extra dimension as well as the two-Higgs-doublet model. An upper limit on the cross-section for $pp\rightarrow G^{*}_{\mathrm {KK}} \rightarrow hh \rightarrow b\bar{b}b\bar{b}$ of 3.2 (2.3) fb is set for a Kaluza–Klein graviton $G^{*}_{\mathrm {KK}}$ mass of 1.0 (1.5) TeV, at the 95 % confidence level. The search for non-resonant Standard Model *hh* production sets an observed 95 % confidence level upper limit on the production cross-section $\sigma (pp \rightarrow hh \rightarrow b\bar{b}b\bar{b})$ of 202 fb, compared to a Standard Model prediction of $\sigma (pp \rightarrow hh \rightarrow b\bar{b}b\bar{b}) = 3.6 \pm 0.5$ fb.

## Introduction

The discovery of a Higgs boson (*h*) [1, 2] at the Large Hadron Collider (LHC) consistent with the predictions of the Standard Model (SM) [3, 4] motivates an enhanced effort to search for new physics via the Higgs sector. Many new physics models predict rates of Higgs boson pair production significantly higher than the SM rate [5–7]. For example, TeV-scale resonances such as the first Kaluza–Klein (KK) excitation of the graviton, $G^{*}_{\mathrm {KK}}$, predicted in the bulk Randall–Sundrum (RS) model [8, 9] or the heavy neutral scalar, *H*, of two-Higgs-doublet models (2HDM) [10] can decay into pairs of Higgs bosons, *hh*. Enhanced non-resonant $pp \rightarrow hh$ production can arise in models such as those with new, light, coloured scalars [11], or direct $t\bar{t}hh$ vertices [12, 13].

ATLAS has carried out a search in the $b\bar{b}\gamma\gamma$ final state [14], setting limits on both resonant (masses between 260 GeVand 500 GeV) and non-resonant Higgs boson pair production. CMS has searched in the multi-lepton and multi-lepton $+$ photons final-states in the context of 2HDM extensions of the Higgs sector [15]. CMS has also searched for narrow resonances in the $b\bar{b}b\bar{b}$ channel [16].

Recent phenomenological studies have demonstrated that despite the fully hadronic final state being subject to a large multijet background, searches for new physics in the $pp \rightarrow hh \rightarrow b\bar{b}b\bar{b}$ process have good sensitivity for both resonant [17, 18] and non-resonant signals [19]. One contributing factor to this sensitivity is the high expected branching ratio for $h \rightarrow b\bar{b}$. The analysis presented in this paper is designed to search for two high-momentum $b\bar{b}$ systems with masses consistent with $m_{h}$, where each $b\bar{b}$ system contains two jets identified as containing *b*-hadrons (the jets are “*b*-tagged”). Compared to a more inclusive $b\bar{b}b\bar{b}$ final-state analysis, this topology has many benefits due to the large required momentum and angular separation between the two $b\bar{b}$ systems: (i) excellent rejection of all backgrounds; (ii) highly efficient triggering using *b*-tagged multijet triggers; and (iii) negligible combinatorial ambiguity in forming Higgs boson candidates.

**Fig. 1:**
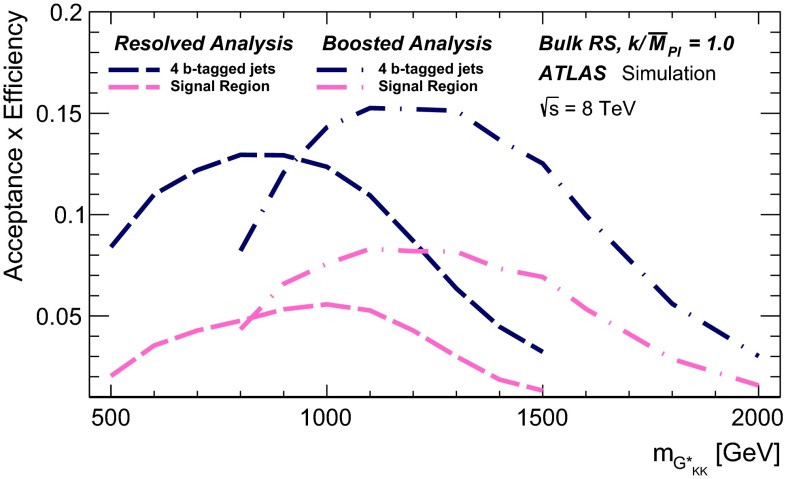
Acceptance times reconstruction and selection efficiency as a function of graviton mass for the bulk RS model, for the resolved and boosted analyses. The shapes of the curves are driven by the separation between the *b*-quarks from the Higgs boson decays and the impact on jet clustering. The requirements are defined in Sects. 4.2 and 5.2

Two Higgs boson reconstruction techniques, which are complementary in their acceptance, are presented. The first – “resolved” – technique reconstructs Higgs boson candidates from pairs of nearby $${\mathrm {anti-}}k_{t}$$ jets [20] with radius parameter $$R = 0.4$$, each *b*-tagged with a multivariate *b*-tagging algorithm [21]. This resolved technique offers good efficiency over a wide range of Higgs boson momenta and so can be used to reconstruct di-Higgs-boson resonances with mass $$m_X$$ in the range between 500 and 1500 GeV. The sensitivity is best for this technique in the range $$500\le m_{X} \lesssim 1100$$ GeV. It can be seen in Fig. 1 however, that the acceptance for four *b*-tagged $${\mathrm {anti-}}k_{t}$$$$R=0.4$$ jets decreases for $$m_X \gtrsim 1200$$ GeV. This loss of acceptance is due to the increased boost of the Higgs boson, which reduces the average separation between the *b*- and $$\bar{b}$$-quarks from the Higgs boson decay, $$\Delta R = \sqrt{(\Delta \eta )^2 + (\Delta \phi )^2}$$, to values below 0.4. This motivates the use of a second – “boosted” – Higgs boson reconstruction technique that maintains acceptance for these higher-mass resonances through the use of jet substructure techniques. The Higgs boson candidate is reconstructed as a single, trimmed [22] $${\mathrm {anti-}}k_{t}$$$$R=1.0$$ jet which must be associated with two *b*-tagged $${\mathrm {anti-}}k_{t}$$$$R = 0.3$$ track-jets [23]. The use of track-jets with a smaller *R* parameter allows Higgs bosons with higher transverse momentum ($$p_{\text {T}}$$) to be reconstructed.

The analysis is performed with the dataset recorded by ATLAS in 2012 at $\sqrt{s}=8$ TeV, corresponding to an integrated luminosity of 19.5 fb$^{-1}$. For the non-resonant search, a counting experiment is performed and the results are interpreted in the context of SM non-resonant Higgs boson pair production. This interpretation is only carried out for the resolved analysis due to its higher sensitivity to such a signal. For the resonant search, a fit to the reconstructed mass spectrum of *hh* candidates is carried out and the results are interpreted in the context of both bulk RS $G^{*}_{\mathrm {KK}}$ (spin-2) and 2HDM CP-even *H* boson (spin-0) production. In the bulk RS model, the fermion and boson fields can propagate in a warped extra dimension, which has a curvature parameter *k*. This benchmark model is investigated with three coupling constant values, $k/\bar{M}_{\mathrm {Pl}} = 1.0,1.5$ and 2.0 ($\bar{M}_{\mathrm {Pl}} = M_{\mathrm {Pl}}/\sqrt{8\pi }$ is the reduced Planck mass), which cover much of the possible parameter space [8]. The 2HDMs considered have CP-conserving scalar potentials (Type-I, Type-II, Lepton-specific and Flipped) [10], in the regime $m_H = m_A = m_{H^{\pm }}$, with the potential parameter that mixes the two Higgs doublets $m_{12}^2~=~m_A^2\tan \beta /(1~+~\tan ^2\beta )$. Interpretations are made as a function of $\tan \beta$ and $\cos (\beta -\alpha )$. The parameter $\tan \beta$ is the ratio of vacuum expectation values of the two Higgs doublets and $\alpha$ is the mixing angle between the two neutral CP-even scalars.

## The ATLAS detector

ATLAS is a multi-purpose particle physics experiment [24] at the LHC. The detector^1^ consists of inner tracking devices surrounded by a superconducting solenoid, electromagnetic and hadronic calorimeters, and a muon spectrometer. The inner tracking system provides charged-particle tracking in the pseudorapidity region $|\eta | < 2.5$ and vertex reconstruction. It consists of a silicon pixel detector, a silicon microstrip tracker, and a straw-tube transition radiation tracker. The system is surrounded by a solenoid that produces a 2 T axial magnetic field. The central calorimeter system consists of a liquid-argon electromagnetic sampling calorimeter with high granularity covering $|\eta | < 3.2$ and a steel/scintillator-tile calorimeter providing hadronic energy measurements in the central pseudorapidity range ($|\eta | < 1.7$). The endcap and forward regions are instrumented with liquid-argon calorimeters for both electromagnetic and hadronic energy measurements up to $|\eta | = 4.9$. The muon spectrometer is operated in a magnetic field provided by air-core superconducting toroids and includes tracking chambers for precise muon momentum measurements up to $|\eta | = 2.7$ and trigger chambers covering the range $|\eta | < 2.4$. A three-level trigger system is used to select interesting events [25]. The Level-1 trigger reduces the event rate to below 75 kHz using hardware-based trigger algorithms acting on a subset of detector information. Two software-based trigger levels, referred to collectively as the High-Level Trigger (HLT), further reduce the event rate to about 400 Hz using information from the entire detector.

## Data and simulation samples

The data sample used in this analysis, after applying data quality requirements that include the availability of *b*-jet triggers, corresponds to an integrated luminosity of $$19.5\pm 0.5$$ fb$$^{-1}$$. The uncertainty in the integrated luminosity ($$2.8~\%$$) is derived following the same methodology as that detailed in Ref. [26]. The data sample is selected by a combination of five triggers requiring multiple jets or *b*-jets, where *b*-jets are identified by a dedicated HLT *b*-tagging algorithm. This combination of triggers is $$>$$$$99.5~\%$$ efficient for signal events passing the offline selection, across the full mass range considered.

Simulated Monte Carlo (MC) event samples are used to model the different signals, as well as the small background contributions from top-quark pair production ($t\bar{t}$) and $Z+$jets events. The dominant multijet background source is estimated directly from data. Signal samples for both models studied are generated with Madgraph v1.5.1 [27, 28], interfaced to Pythia  v8.175 [29] for parton showering, hadronization and underlying-event simulation. The Higgs boson mass is set to 125 GeV. The CTEQ6L1 [30] leading-order (LO) parton distribution functions (PDFs) are used. Table 1 provides the calculated cross-sections and widths for different signal model parameters. The bulk RS model predictions are calculated at LO using Madgraph. The 2HDM prediction corresponds to the cross-section for gluon-fusion production plus *b*-associated production plus vector-boson-fusion production. The gluon-fusion cross-section is calculated using SusHi v1.3.0 [31–36] at next-to-next-to-leading-order (NNLO) accuracy in QCD. For *b*-associated production, an empirical matching of the four- and five-flavour scheme is used [37]. The four-flavour cross-section is calculated at next-to-leading-order (NLO) accuracy in QCD following Refs. [38, 39], while the five-flavour cross-section is calculated at NNLO in QCD using SusHi. The vector-boson-fusion cross-section at NNLO accuracy in QCD and NLO in electroweak is taken from Ref. [40] and corrected by a multiplicative factor of $\cos ^2(\beta - \alpha )$ [10]. The 2HDM branching ratios are calculated using 2HDMC v1.6.4 [41].

**Table 1:** Computed production cross-sections times branching ratio $\sigma \left( pp \rightarrow X \rightarrow hh \rightarrow b\bar{b}b\bar{b}\right)$ and total widths for selected resonance pole mass values. The bulk RS model predictions are obtained with $k/\bar{M}_{\mathrm {Pl}}$  = 1.0; both cross-section and width grow as the square of $k/\bar{M}_{\mathrm {Pl}}$. The 2HDM predictions are for a Type-II model with $\cos \left( \beta -\alpha \right) =-0.2$ and $\tan \beta = 1$

Model	Mass [GeV]	$\sigma \,\times$ B [fb]	$\Gamma$ [GeV]
Bulk RS	1000	1.47	55
Bulk RS	1500	0.085	90
2HDM	1000	5.54	130
2HDM	1500	0.330	332

For the $$G^{*}_{\mathrm {KK}}$$$$ \rightarrow hh \rightarrow b\bar{b}b\bar{b}$$  signal, three sets of MC samples are generated for each of the three coupling values, $$k/\bar{M}_{\mathrm {Pl}}$$$$=$$ 1.0, 1.5 and 2.0. The variation in these couplings alters both the total $$G^{*}_{\mathrm {KK}}$$ production cross-section and its width. Samples cover a range of $$G^{*}_{\mathrm {KK}}$$ masses $$500\le m_{G^{*}_{\mathrm {KK}}} \le 2000$$ GeV. For the $$H \rightarrow hh \rightarrow b\bar{b}b\bar{b}$$  signal, samples are generated covering the range $$500\le m_{H}\le 1500$$ GeV. Since the width of *H*, $$\Gamma _H$$, varies non-trivially with the 2HDM parameters, the samples are generated with fixed $$\Gamma _H = 1$$ GeV. In order to make 2HDM interpretations of the results obtained with these fixed width samples, they are corrected to account for the true resonance width at each point in parameter space, as described in Sect. 6.

Non-resonant SM $pp \rightarrow hh \rightarrow b\bar{b}b\bar{b}$ events are generated using the exact form factors for the top loop, taken from HPAIR [5, 42]. The cross-section is defined as the inclusive cross-section. The gluon-fusion production cross-section at NNLO in QCD from Ref. [7] is used, summed with the NLO predictions for vector-boson-fusion, top-pair-associated and vector-boson-associated production from Ref. [43]. The resulting cross-section is $\sigma (pp\rightarrow hh\rightarrow b\bar{b}b\bar{b}) = 3.6\pm 0.5$ fb, where the uncertainty term includes the effects of uncertainties in the renormalization and factorization scale, PDFs, $\alpha _S$ and $Br(H\rightarrow b\bar{b})$.

The $t\bar{t}$  background sample is generated using Powheg-box v1.0 [44–47] interfaced to Pythia  v6.426 [48], with the top mass fixed to 172.5 GeVand the CT10 [49] NLO PDF set. The NNLO+NNLL prediction of 253 pb for the $t\bar{t}$  cross-section [50–55] is used for normalization. Single-top background is negligible.

The $Z+$jets sample is generated using Sherpa v1.4.3 [56] with the CT10 PDF set and the *Z* boson decaying to two *b*-quarks. The $Z+$jets cross-section is taken from an NLO Powheg-box v1.0 [57] plus Pythia  v8.165 prediction, which is found to agree well with measurements in the boosted regime [58].

The generated MC events are processed with the GEANT4-based [59] ATLAS detector simulation [60]. Effects of multiple proton–proton interactions (pile-up) are simulated using Pythia  v8.1 with the CTEQ6L1 PDF set and the AU2 tune [61]. The simulated events are weighted so that the distribution of the average number of interactions per bunch-crossing matches that in the data. The same reconstruction software is used to process both the data and the simulated samples.

Table 2 summarizes the various event generators and PDF sets, as well as parton shower and hadronization software used for the analyses presented in this paper.

**Table 2:** Summary of MC event generators, PDF sets, parton shower and hadronization used in the analysis for both signal and background processes. * Madgraph was modified [62] to use the exact top-loop form-factors from HPAIR [5, 42] for the gluon-fusion production process

Model/process	Generator	PDF	Parton Shower/hadron
Bulk RS: $pp\rightarrow G^{*}_{\mathrm {KK}} \rightarrow hh \rightarrow b\bar{b}b\bar{b}$	Madgraph v1.5.1 [27, 28]	CTEQ6L1 [30]	Pythia v8.175 [29]
2HDM: $pp\rightarrow H \rightarrow hh \rightarrow b\bar{b}b\bar{b}$	Madgraph v1.5.1 [27]	CTEQ6L1 [30]	Pythia v8.175 [29]
SM: $pp \rightarrow hh \rightarrow b\bar{b}b\bar{b}$	Madgraph v1.5.1 [27, 62]*	CTEQ6L1 [30]	Pythia v8.175 [29]
$t\bar{t}$	Powheg v1.0 [44, 45]	CT10 [49]	Pythia v6.426 [48]
$Z+$jets	Sherpa v1.4.3 [56]	CT10 [49]	Sherpa v1.4.3 [56]

## Resolved analysis

### Event reconstruction

Jets are reconstructed from topological clusters of calorimeter cell energy deposits at the electromagnetic scale [63] using the ${\mathrm {anti-}}k_{t}$ jet clustering algorithm, with a radius parameter of $R = 0.4$. The effects of pile-up on jet energies are accounted for by a jet-area-based correction [64]. The jets are then calibrated using $p_{\text {T}}$- and $\eta$-dependent calibration factors based on MC simulations and the combination of several in situ techniques applied to data [65]. Following this, the jets undergo Global Sequential calibration [63] which reduces flavour-dependent differences in calorimeter response. If a muon with $p_{\text {T}} > 4$ GeV and $|\eta | < 2.5$ is found within a cone of $\Delta R=0.4$ around the jet axis, the four-momentum of the muon is added to that of the jet (after correcting for the expected energy deposited by the muon in the calorimeter). Such muons are reconstructed by combining measurements from the inner tracking and muon spectrometer systems, and are required to satisfy tight muon identification quality criteria [66]. Jets with a significant energy contribution from pile-up interactions [67] are removed using tracking information. For jets with $p_{\text {T}}$  $<$ 50 GeV and $|\eta | < 2.4$, the $p_{\text {T}}$  sum of tracks matched to the jet is calculated and it is required that at least 50 % of this $p_{\text {T}}$  sum is due to tracks originating from the primary vertex.^2^

Jets with $|\eta | < 2.5$ are *b*-tagged using the properties of the tracks associated with them, the most important being the impact parameter (defined as the track’s distance of closest approach to the primary vertex in the transverse plane) of each track, as well as the presence and properties of displaced vertices. The MV1 *b*-tagging algorithm [21] used in this analysis combines the above information using a neural network and is configured to achieve an efficiency of 70 % for tagging *b*-jets,^3^ with a charm-jet rejection of approximately 5 and a light-quark or gluon jet rejection of around 140, as determined in an MC sample of $t\bar{t}$  events. The *b*-tagging efficiency in the simulation is scaled to reproduce the one measured in data [68].

### Selection

**Fig. 2:**
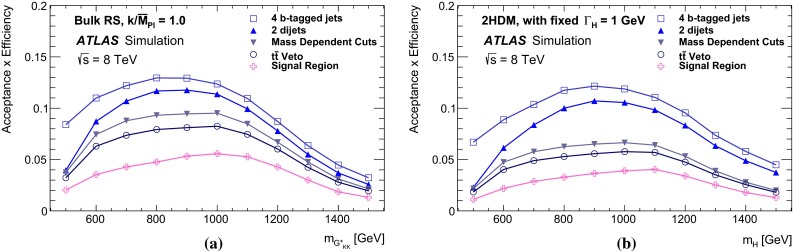
The selection efficiency as a function of resonance mass at each stage of the event selection for **a** $G^{*}_{\mathrm {KK}}$ $\rightarrow hh \rightarrow b\bar{b}b\bar{b}$ events and **b** $H \rightarrow hh \rightarrow b\bar{b}b\bar{b}$ events in the resolved analysis

The combined acceptance times efficiency ($$A\times \varepsilon $$) at different stages of the event selection is shown in Fig. 2 as a function of resonance mass for the resonant signal models. The event selection begins with the requirement of at least four *b*-tagged jets, each with $$p_{\text {T}} > 40$$ GeV (shown in Fig. 2 as “4 *b*-tagged jets”). SM non-resonant Higgs boson pair production has a softer Higgs boson $$p_{\text {T}}$$ spectrum than the $$m_X = 500~{\mathrm {\ GeV}}{}$$ resonances, resulting in a lower $$A\times \varepsilon = 4.9~\%$$ for this requirement. The four highest-$$p_{\text {T}}$$  *b*-tagged jets are then used to form two dijet systems, demanding that the angular distance, $$\Delta R$$, between the jets in each of the dijets is smaller than 1.5. The transverse momentum of the leading (in $$p_{\text {T}}$$) dijet system, $$p_{\text {T}} ^{\mathrm {lead}}$$, is required to be greater than 200 GeV, while the subleading dijet system must have $$p_{\text {T}} ^{\mathrm {subl}}$$$$> 150$$ GeV. In the rare case that a jet could be used to create more than one dijet which satisfies the above kinematic requirements, the dijet with the highest mass is chosen. Thus two unique dijet systems, with no jets in common, are selected (shown as “2 dijets” in Fig. 2). For SM non-resonant Higgs boson pair production, after this requirement, $$A\times \varepsilon = 1.2~\%$$. The impact of different decay kinematics can be seen by comparing Fig. 2b to Fig. 2a: the decay of spin-0 *H* bosons gives a softer Higgs boson $$p_{\text {T}}$$ spectrum than in the case of the spin-2 $$G^{*}_{\mathrm {KK}}$$ decay (due to the differing angular distributions of hh), resulting in lower acceptance for these kinematic requirements at low resonance mass.

The resolved analysis considers a large range of resonance masses, $500\le m_X \le 1500$ GeV. Due to the differing kinematics, the optimal selection for low-mass resonances differs from the optimum for higher masses. To increase the analysis sensitivity, three requirements which vary with the reconstructed resonance mass are used. These selection requirements are optimized simultaneously, by performing a three-dimensional scan of threshold values, using the statistical-only exclusion limit (Sect. 6) as the objective function. There are mass-dependent requirements (shown in Fig. 2 as “MDC”) on the minimum $p_{\text {T}}$ of the leading and subleading dijets as well as on the maximum difference in pseudorapidity, $|\Delta \eta _{\mathrm {dijets}}|$, between them. These requirements are written in terms of the four-jet mass $m_{\mathrm {4j}}$ expressed in GeV:

$$\begin{aligned} p_{\text {T}} ^{\mathrm {lead}} > {\left\{ \begin{array}{ll} 400\,{\mathrm {\ GeV}}{}&{} \mathrm {if}\ m_{\mathrm {4j}} > 910\,{\mathrm {\ GeV}}{}, \\ 200\,{\mathrm {\ GeV}}{}&{} \mathrm {if}\ m_{\mathrm {4j}} < 600\,{\mathrm {\ GeV}}{}, \\ 0.65m_{\mathrm {4j}}- 190\,{\mathrm {\ GeV}}{}&{} \mathrm {otherwise}, \end{array}\right. } \end{aligned}$$

$$\begin{aligned} p_{\text {T}} ^{\mathrm {subl}} > {\left\{ \begin{array}{ll} 260\,{\mathrm {\ GeV}}{}&{} \mathrm {if}\ m_{\mathrm {4j}} > 990\,{\mathrm {\ GeV}}{}, \\ 150\,{\mathrm {\ GeV}}{}&{} \mathrm {if}\ m_{\mathrm {4j}} < 520\,{\mathrm {\ GeV}}{}, \\ 0.235m_{\mathrm {4j}} + 28\,{\mathrm {\ GeV}}{}&{} \mathrm {otherwise}, \end{array}\right. } \end{aligned}$$

$$\begin{aligned} \left| \Delta \eta _{\mathrm {dijets}}\right| < {\left\{ \begin{array}{ll} 1 &{} \mathrm {if}\ m_{\mathrm {4j}} < 820\,{\mathrm {\ GeV}}{}, \\ 1.55\times 10^{-3}m_{\mathrm {4j}}- 0.27 &{} \mathrm {otherwise}. \end{array}\right. } \end{aligned}$$

The different $m_{\mathrm {4j}}$ thresholds shown above are chosen to obtain a continuously varying set of requirements. The requirement on $\left| \Delta \eta _{\mathrm {dijets}}\right|$ leads to a lower acceptance for *H* compared to $G^{*}_{\mathrm {KK}}$ for $m_{X} \ge 700$ GeV because of the effect of the boson spin on the angular distribution of its decay products.

After selecting two dijets that satisfy the mass-dependent criteria, $t\bar{t}$ constitutes approximately 10 % of the total background. This $t\bar{t}$  background predominantly comprises events where both top quarks decayed hadronically. These hadronic decays lead to three jets for each top quark: one *b*-jet directly from the top decay and two from the decay of the *W* boson. Since the probability to mis-tag charm-jets is much higher than the probability to mis-tag light-jets, in the majority of cases the dijet is formed from the *b*-jet and a charm-jet from the decay of the *W* boson. In order to reduce the $t\bar{t}$  background, jets not already used in the formation of the two dijets (“extra jets”) in the event are used to reconstruct *W* boson and top quark candidates by combining them with each of the dijets. These extra jets are required to have $p_{\text {T}} > 30$ GeV, $|\eta | < 2.5$, and $\Delta R<1.5$ relative to the dijet. The *W* boson candidates are reconstructed by adding the four-momentum of each of the possible extra jets to the four-momentum of the jet in the dijet system with the lowest probability of being a *b*-jet according to the multivariate *b*-tagging algorithm. Top quark candidates are then reconstructed by summing the dijets with each of the extra jets. The compatibility with the top quark decay hypothesis is then determined using the variable:

$$\begin{aligned} X_{tt}\,=\,\sqrt{\left( \frac{m_{W}\,-\,\tilde{m}_{W}}{\sigma _{m_{W}}}\right) ^2 + \left( \frac{m_{t}\,-\,\tilde{m}_{t}}{\sigma _{m_{t}}}\right) ^2}, \end{aligned}$$

where $$m_{W}$$ and $$m_t$$ are the invariant masses of the *W* boson and top quark candidates, $$\sigma _{m_{W}} = 0.1\, m_{W}$$, $$\sigma _{m_{t}} = 0.1\, m_{t}$$, $$\tilde{m}_{W} = 80.4$$ GeV and $$\tilde{m}_{t} = 172.5$$ GeV. The values of $$\sigma _{m_{W}}$$ and $$\sigma _{m_{t}}$$ reflect the dijet and three-jet system mass resolutions. If either dijet in an event has $$X_{tt} < 3.2$$ for any possible combination with an extra jet, the event is rejected. This requirement reduces the $$t\bar{t}$$  background by $$\sim $$$$60~\%$$, whilst retaining $$\sim $$$$90~\%$$ of signal events (shown as “$$t\bar{t}$$ Veto” in Fig. 2).

The event selection criteria described above are collectively referred to as the “4-tag” selection requirements. These requirements select 1891 events.

**Fig. 3:**
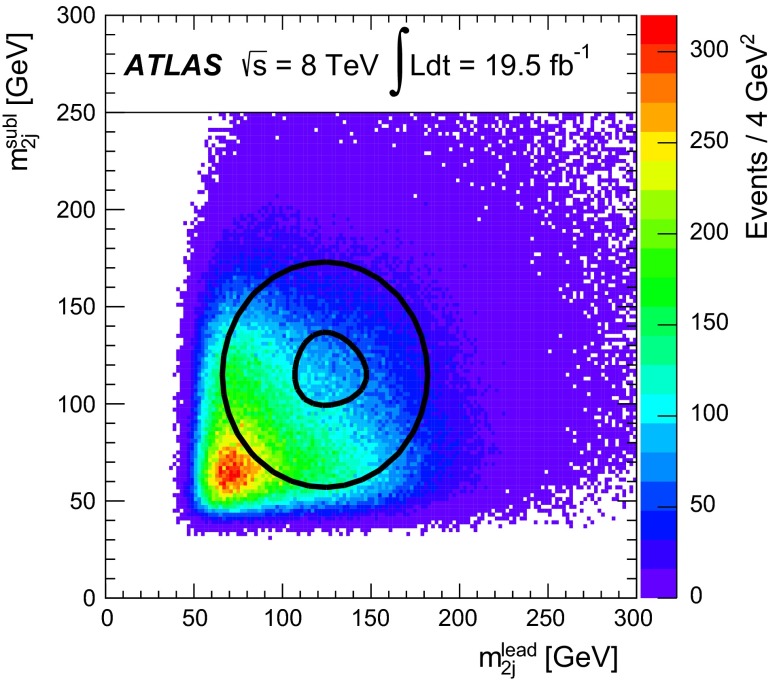
The distribution of the subleading dijet mass, $m_{\mathrm {2j}}^{\mathrm {subl}}$, vs the leading dijet mass, $m_{\mathrm {2j}}^{\mathrm {lead}}$, for the 2-tag data sample used to model the multijet background in the resolved analysis. The signal region is the area surrounded by the *inner black contour line*, centred on $m_{\mathrm {2j}}^{\mathrm {lead}} =124$ GeV, $m_{\mathrm {2j}}^{\mathrm {subl}} =115$ GeV. The control region is the area inside the *outer black contour line*, excluding the signal region. The sideband region is the area outside the *outer contour line*

Following the 4-tag selection, a requirement on the leading and subleading dijet masses ($m_{\mathrm {2j}}^{\mathrm {lead}}$ and $m_{\mathrm {2j}}^{\mathrm {subl}}$, respectively) is used to define the signal region. The central value of this region corresponds to the median values of the narrowest dijet mass intervals that contain 90 % of the MC signal (these were found to be stable with resonance mass). The definition of the signal region is

1 $$\begin{aligned} X_{hh}\,=\,\sqrt{\left( \frac{m_{\mathrm {2j}}^{\mathrm {lead}} \,-\,124\,{\mathrm {\ GeV}}{}}{0.1\,m_{\mathrm {2j}}^{\mathrm {lead}}}\right) ^2 + \left( \frac{m_{\mathrm {2j}}^{\mathrm {subl}} \,-\,115\,{\mathrm {\ GeV}}{}}{0.1\,m_{\mathrm {2j}}^{\mathrm {subl}}}\right) ^2}, \end{aligned}$$

where the $0.1\,m_{\mathrm {2j}}$ terms represent the widths of the leading and subleading dijet mass distributions. The signal region is defined as $X_{hh} < 1.6$. This corresponds to the kinematical requirements illustrated by the inner region in Fig. 3, albeit with data from the 2-tag sample shown. It is optimized to maximize the expected sensitivity of the search. The acceptance times efficiency of the full selection, including this signal region requirement, is shown in Fig. 2 as “Signal Region”. For SM non-resonant Higgs boson pair production, the full selection has an $A\times \varepsilon = 0.60~\%$.

The final step of the Higgs boson pair resonant production search is to perform a fit to the four-jet mass $$m_{\mathrm {4j}}$$ in the signal region. The sensitivity of this fit is increased by improving the $$m_{\mathrm {4j}}$$ resolution in this region, using the constraint that the two dijet masses should equal the Higgs boson mass, i.e. $$m_{\mathrm {2j}}^{\mathrm {lead}} = m_{\mathrm {2j}}^{\mathrm {subl}} = m_h$$. To this end, each dijet four-momentum is multiplied by a correction factor $$\alpha _{\mathrm {dijet}} = m_h/m_{\mathrm {dijet}}$$. This leads to an improvement of $$\sim $$$$30~\%$$ in the signal $$m_{\mathrm {4j}}$$ resolution – with a significant reduction of the low-mass tails caused by energy loss – with little impact on the background.

### Background estimation

After the 4-tag selection described above, about 95 % of the remaining background in the signal region is expected to originate from multijet events, which are modelled using data. The remaining $$\sim $$$$5~\%$$ of the background is $$t\bar{t}$$  events. The $$t\bar{t}$$  yield is determined from data, while the $$m_{\mathrm {4j}}$$  shape is taken from MC simulation. The $$Z+$$jets contribution is $$<1$$ % of the total background and is modelled using MC simulation. The background from all other sources – including processes featuring Higgs bosons – is negligible.

#### Multijet background

The multijet background is modelled using an independent data sample selected by the same trigger and selection requirements as described in Sect. 4.2, except for the *b*-tagging requirement: only one of the two selected dijets has to be formed from *b*-tagged jets, while the other dijet can be formed from jets that are not *b*-tagged. This “2-tag” selection yields a data sample comprising 485377 events, 98 % of which are multijet events and the remaining 2 % are $t\bar{t}$. The predicted contamination by the signal is negligible.

This 2-tag sample is normalized to the 4-tag sample and its kinematical distributions are corrected for differences introduced by the additional *b*-tagging. These differences arise because the *b*-tagging efficiency as well as the charm- and light-jet rejection vary as a function of jet $p_{\text {T}}$ and $\eta$, the various multijet processes contribute in different fractions, and the fraction of events passed by each trigger path changes. The normalization and kinematic corrections are determined using a signal-free sideband region of the $m_{\mathrm {2j}}^{\mathrm {lead}}$–$m_{\mathrm {2j}}^{\mathrm {subl}}$ plane, in dedicated samples collected without mass-dependent requirements, which increases the statistical precision of the kinematic corrections. The resulting background model is verified and the associated uncertainties are estimated using a control region. The sideband and control regions are shown in Fig. 3. The sideband region is defined as: $\sqrt{(m_{\mathrm {2j}}^{\mathrm {lead}}- 124~{\mathrm {\ GeV}}{})^2 + (m_{\mathrm {2j}}^{\mathrm {subl}}- 115~{\mathrm {\ GeV}}{})^2}~>~58~\mathrm {{\mathrm {\ GeV}}{}}$, while the control region is defined as the region between the signal and sideband regions. These definitions are chosen to be orthogonal to the signal region and to give approximately equal event yields in the sideband and control regions.

The normalization of the multijet background prediction is set by scaling the number of events in each region of the 2-tag sample by the following factor, $\mu _{\mathrm {QCD}}$, calculated in the sideband region:

2 $$\begin{aligned} \mu _{\mathrm {QCD}} = \frac{N^{\mathrm {4-tag}}_{\mathrm {QCD}}}{N^{\mathrm {2-tag}}_{\mathrm {QCD}}} = \frac{N^{\mathrm {4-tag}}_{\mathrm {data}} - N^{\mathrm {4-tag}}_{t\bar{t}} - N^{\mathrm {4-tag}}_{Z}}{N^{\mathrm {2-tag}}_{\mathrm {data}}- N^{\mathrm {2-tag}}_{t\bar{t}} - N^{\mathrm {2-tag}}_{Z}}, \end{aligned}$$

where $N_{\mathrm {data}}^{\mathrm {2-/4-tag}}$ is the number of events observed in the sideband region in the 2- or 4-tag data sample, respectively. The yields $N_{t\bar{t}}^{\mathrm {2-/4-tag}}$ and $N_Z^{\mathrm {2-/4-tag}}$ are the estimated number of $t\bar{t}$ and $Z+$jets events in the 2-/4-tag selected sideband region. The $t\bar{t}$  normalization is estimated from data, as described in Sect. 4.3.2, while the $Z+$jets is estimated using MC simulation.

To predict the distributions of the multijet background in each region, the predicted $t\bar{t}$ and $Z+$jets 2-tag distributions are first subtracted from the 2-tag data sample distribution before the distribution is scaled by $\mu _{\mathrm {QCD}}$.

The correction for the kinematic differences between 2-tag and 4-tag samples is performed by reweighting events in the 2-tag sample. The weights are derived in the sideband region from linear fits to the ratio of the total background model to data for three kinematic distributions which are found to have the largest disagreement between 2-tag and 4-tag events: the leading dijet $p_{\text {T}}$, the $\Delta R$ separation between the jets in the subleading dijet, and the $\Delta R$ separation between the two dijets. The reweighting is done using one-dimensional distributions, but is iterated so that correlations between the three variables are approximately accounted for. Three iterations are found to be sufficient. After the correction process, there is good agreement between the background model and sideband region data in kinematic variables that were not explicitly corrected. Systematic uncertainties in the normalization and shape of the multijet background model in the signal region are assessed using control-region data, as described in Sect. 4.4.

#### $t\bar{t}$ background

The $t\bar{t}$  background is described using a hybrid model: the normalization is derived from data in a $t\bar{t}$  control sample, while the shape is taken from MC simulation because there are too few events in the $t\bar{t}$  control sample to describe the shape precisely enough.

The $$t\bar{t}$$  control sample is formed from events which pass the 4-tag selection, except for the top veto, which is reversed: if either of the dijets fails the top veto, the event enters the $$t\bar{t}$$  control sample. This selection leads to a sample of 41 events within the signal region of the $$t\bar{t}$$  control sample, of which $$\sim $$$$50~\%$$ are $$t\bar{t}$$ and $$\sim $$$$50~\%$$ multijet. The multijet background component is estimated using the same methods as used for the nominal selection, but with a wider control region in order to reduce the sideband region $$t\bar{t}$$  fraction. After subtracting the multijet background, the $$t\bar{t}$$  control sample yield is then extrapolated to predict the $$t\bar{t}$$  yield in the nominal signal region, $$N_{t\bar{t}}$$, using the following equation:

3 $$\begin{aligned} N_{t\bar{t}} = \frac{\epsilon _{t}^{2}}{1-\epsilon _{t}^{2}} \times N_{t\bar{t}}^{\mathrm {CS}}, \end{aligned}$$

where $$N_{t\bar{t}}^{\mathrm {CS}}$$ is the number of events in the signal region, after subtraction of the multijet background, within the $$t\bar{t}$$  control sample, and $$\epsilon _{t}$$ is the efficiency for a selected dijet in a $$t\bar{t}$$  event to pass the top veto. This equation relies on the assumption that the $$\epsilon _{t}$$ of each dijet in the event is uncorrelated, an assumption validated in $$t\bar{t}$$  MC simulation. The $$\epsilon _{t}$$ is measured using an independent “semileptonic $$t\bar{t}$$ ” data sample that has a high $$t\bar{t}$$  purity. Events in this sample are selected by requiring one dijet candidate to pass the nominal selection with $$p_{\text {T}} $$$$>$$$$150$$ GeV and one “leptonic top-quark” candidate. The leptonic top quark candidate is defined using a reconstructed muon and one *b*-tagged jet. This *b*-tagged jet is required to be distinct from jets in the dijet candidate, and the muon is required to have $$p_{\text {T}}$$$$>$$25 GeV, be isolated, and fall within a cone of radius 1.2 around the *b*-tagged jet. The leptonic top quark candidate is required to have $$p_{\text {T}}$$  $$>$$180 GeV, where the leptonic top $$p_{\text {T}}$$ is defined as the vector sum of the *b*-jet $$p_{\text {T}}$$, the muon $$p_{\text {T}}$$, and the missing transverse momentum in the event. The latter is reconstructed from energy deposits in the calorimeter, including corrections for identified jets, electrons and muons. The $$t\bar{t}$$  veto efficiency is then measured as the fraction of the reconstructed dijet candidates which passed the $$t\bar{t}$$  veto, yielding $$\epsilon _{t} = $$ 0.53 $$\pm $$ 0.03 (stat.) $$\pm $$ 0.05 (syst.). The systematic uncertainty in $$\epsilon _{t}$$ is assigned to cover potential differences between $$\epsilon _{t}$$ as measured in the semileptonic $$t\bar{t}$$  sample and $$\epsilon _{t}$$ in the full 4-tag selection, where the method is applied in $$t\bar{t}$$  MC simulation to evaluate such differences. The measured $$\epsilon _{t}$$ agrees well with the corresponding semileptonic $$t\bar{t}$$  MC prediction of 0.54.

Equation (3) gives a data-driven $t\bar{t}$  background prediction of $5.2 \pm 2.6$ events in the signal region after the full selection. The uncertainty is dominated by the statistical uncertainty in $N_{t\bar{t}}^{\mathrm {CS}}$, with a smaller contribution from the uncertainty in $\epsilon _{t}$.

Due to the limited number of events in the $t\bar{t}$  control sample, the $m_{\mathrm {4j}}$  shape of the $t\bar{t}$  background is modelled using MC simulation. However, despite the use of a large $t\bar{t}$  sample, very few events pass the full 4-tag selection. Therefore, the $t\bar{t}$  shape is derived from MC simulation using the “2-tag” selection, with a systematic uncertainty assigned to cover differences between the 2-tag and 4-tag $m_{\mathrm {4j}}$ distributions.

### Systematic uncertainties

Two classes of systematic uncertainties are evaluated: those affecting the modelling of the signal and those affecting the background prediction.

The signal modelling uncertainties comprise: theoretical uncertainties in the acceptance, uncertainties in the jet energy scale (JES) and resolution (JER), and uncertainties in the *b*-tagging efficiency.

The theoretical uncertainties considered arise from initial- and final-state radiation modelling (ISR and FSR), PDF uncertainties and uncertainty in the LHC beam energy. These are estimated using particle-level samples generated using the same generator configurations as the nominal signal samples but with appropriate variations, assessing the difference in yields after the full analysis selection. The ISR and FSR uncertainty is evaluated by varying the relevant parton shower parameters in Pythia 8. The PDF uncertainty is estimated by taking the maximum difference between the predictions when using MSTW2008nlo [69], NNPDF2.3 [70] and CTEQ6L1. The uncertainty due to the beam energy is determined by varying coherently the energy of each beam by $$\pm $$$$26.5{\mathrm {\ GeV}}{}$$ [71] in the simulation. Only FSR has a significant impact on the acceptance, leading to a $$\pm $$$$1.0~\%$$ theoretical modelling acceptance uncertainty.

The JES systematic uncertainty is evaluated using 15 separate and orthogonal uncertainty components, which allow for the correct treatment of correlations across the kinematic bins [65]. The JER uncertainty is evaluated by smearing jet energies according to the systematic uncertainties of the resolution measurement performed with data [65]. For *b*-jets with $p_{\text {T}}$  $<$ 300 GeV the uncertainty in the *b*-tagging efficiency is evaluated by propagating the systematic uncertainty in the measured tagging efficiency for *b*-jets [68], which ranges from 2 % to 8 % depending on *b*-jet $p_{\text {T}}$ and $\eta$. However, for the higher resonance masses considered in this analysis, there are a significant number of events containing at least one *b*-jet with $p_{\text {T}}$  $>$ 300 GeV. The systematic uncertainties in the tagging efficiencies of these jets are derived from MC simulation and are larger, reaching 24 % for $p_{\text {T}}$ $>$ 800 GeV.

Systematic uncertainties in the normalization and shape of the multijet background model are assessed in the control region. Table 3 shows the estimated background yields in the control and sideband regions. The control region background prediction agrees with the observed data within the data statistical uncertainty of $\pm 3.5~\%$. To further test the robustness of the background estimation and the assumptions behind it, predictions are made with different sideband and control region definitions and different *b*-tagging requirements on the 2-tag sample. Redefinitions of the sideband and control region changed the kinematic composition of these regions, enhancing the sideband region in either high mass or low mass dijets and therefore altering the kinematic corrections that are applied. These variations induce a maximum change of $\pm 6~\%$ in the estimated multijet yield and so the uncertainty is set to this value. Different *b*-tagging requirements on the *b*-tagged dijet in the 2-tag sample are used in order to change the composition of the sample and to vary the degree of *b*-tagging-related kinematic bias. No additional uncertainty is required.

**Table 3:** The number of events in data and predicted background events after applying the mass-dependent requirements in preselection and in the sideband and control regions for the resolved analysis. The uncertainties are purely statistical. The $t\bar{t}$  yield in this table, in contrast to the final result, is estimated using MC simulation

Sample	Sideband region	Control region
Multijet	907 $\pm$ 3	789 $\pm$ 3
$t\bar{t}$	25.5 $\pm$ 0.3	57.5 $\pm$ 0.4
*Z*+jets	14 $\pm$ 1	20 $\pm$ 1
Total	945 $\pm$ 3	867 $\pm$ 3
Data	952	852

**Fig. 4:**
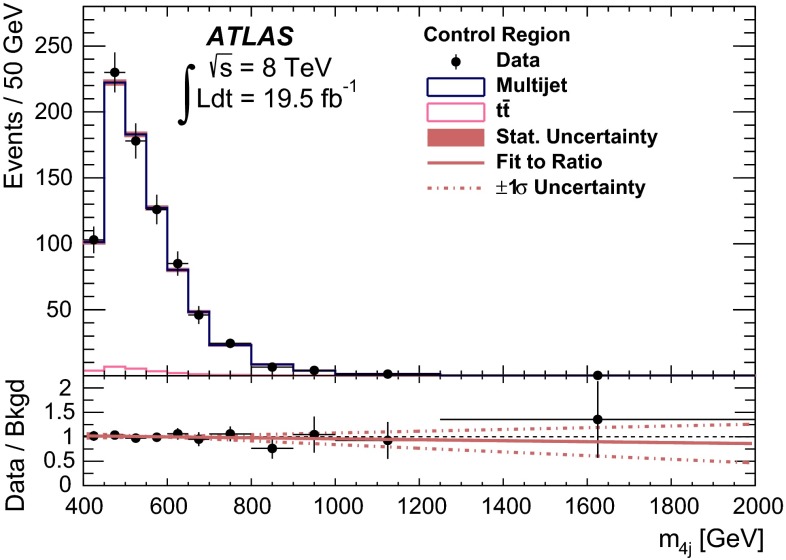
The four-jet mass, $m_{\mathrm {4j}}$, distribution in the control region (*points*) for the resolved analysis, compared to the predicted background (*histograms*). The *small filled blocks* represent the statistical uncertainty in the total background estimate. The *bottom panel *shows the first-order polynomial fit to the data-to-background ratio of the $m_{\mathrm {4j}}$  distribution that is used to fix the multijet shape systematic uncertainty. The *dashed lines* show the $\pm$ $1\sigma$ uncertainties in the two fitted parameters

The uncertainty in the description of the multijet $m_{\mathrm {4j}}$ distribution is determined by comparing the total background prediction to data in the control region, as shown in Fig. 4. Good agreement in the shape is observed and a straight line fit to the ratio of the distributions gives a slope consistent with zero. This fit, along with its uncertainties, is shown in the bottom panel of Fig. 4. The uncertainty in the multijet background shape is defined using the uncertainty in the fitted slope.

The uncertainty in the $$t\bar{t}$$  normalization is dominated by the statistical uncertainty of the yield in the $$t\bar{t}$$  control sample, with a subdominant contribution from the uncertainties in the top veto efficiency, $$\epsilon _{t}$$, leading to a total uncertainty of $$\pm 50~\%$$. The uncertainty in the MC-derived $$t\bar{t}$$$$m_{\mathrm {4j}}$$ distribution is dominated by the uncertainty associated with using the shape after the 2-tag selection, rather than the 4-tag selection. This uncertainty is assessed by comparing the 2-tag to 4-tag MC predictions in the signal region. A straight line fit to the ratio of the normalized distributions is made and used to define a shape uncertainty in the same way as the multijet background. Due to the large statistical uncertainties of the 4-tag $$t\bar{t}$$  sample, the assigned shape uncertainty is large: $$\sim $$$$30~\%$$ and $$\sim $$$$100~\%$$ in the event yield at $$m_{\mathrm {4j}}$$ = 400 GeV and $$1500~{\mathrm {\ GeV}}{}$$, respectively.

**Fig. 5:**
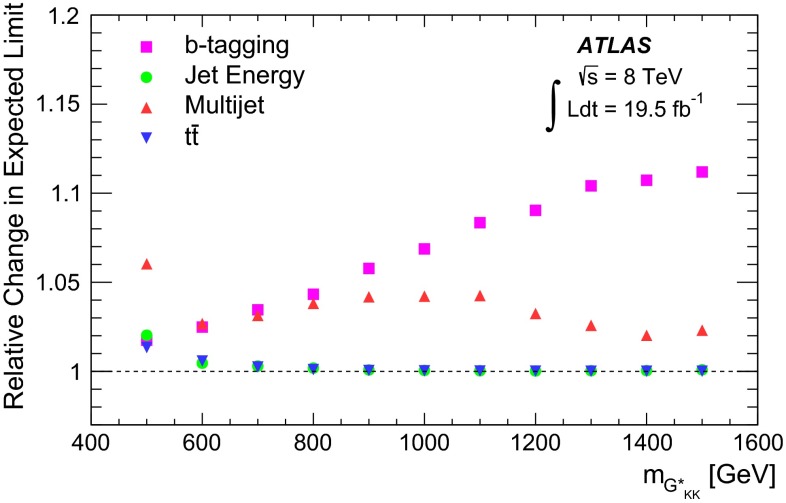
The individual impact of the systematic uncertainties considered in the resolved analysis on the expected $\sigma \left( pp \rightarrow G^{*}_{\mathrm {KK}} \rightarrow hh \rightarrow b\bar{b}b\bar{b}\right)$ 95 % confidence level exclusion limit, as a function of graviton mass. The calculation of the expected limit is described in Sect. 6. Only the mass-dependent uncertainties are shown. The impact is the ratio of the limit calculated using all systematic uncertainties sources to the limit calculated using all systematic uncertainty sources excluding those under investigation

Table 4 shows the relative impact of the uncertainties in the event yields. Figure 5 shows the relative impact on the expected limit for $\sigma \left( pp \rightarrow G^{*}_{\mathrm {KK}} \rightarrow hh \rightarrow b\bar{b}b\bar{b}\right)$. The calculation of the expected limit is described in Sect. 6. It can be seen that for resonance masses below 700 GeV, the effect on the limit is dominated by the multijet description, with a small contribution from the $t\bar{t}$ background since both backgrounds are predominately at low mass. Above $m_X = 700$ GeV, the uncertainty associated with the modelling of the *b*-tagging efficiency has the largest impact, since the larger high-$p_{\text {T}}$ uncertainties have an increasingly important effect with mass.

**Table 4:** Summary of systematic uncertainties (expressed in percent) in the total background and signal event yields, in the signal region of the resolved analysis. Signal yield uncertainties are provided for non-resonant SM Higgs boson pair production and three resonances with $m=1000~{\mathrm {\ GeV}}{}$: a $G^{*}_{\mathrm {KK}}$ with $k/\bar{M}_{\mathrm {Pl}}$ $=$ 1, another with $k/\bar{M}_{\mathrm {Pl}}$ $=$ 2, and *H* with fixed $\Gamma _H = 1~{\mathrm {\ GeV}}{}$

Source	Bkgd	SM *hh*	$G^{*}_{\mathrm {KK}}$		*H*
			$\frac{k}{\bar{M}_{\mathrm {Pl}}}$ $=$ 1	$\frac{k}{\bar{M}_{\mathrm {Pl}}}$ $=$ 2	
Luminosity	–	2.8	2.8	2.8	2.8
JER	–	4.5	1.1	1.1	2.0
JES	–	7	1.8	1.3	3.4
*b*-tagging	–	12	22	21	22
Theoretical	–	1.0	1.1	1.1	1.1
Multijet	6.0	–	–	–	–
$t\bar{t}$	3.0	–	–	–	–
Total	6.7	15	22	22	23

### Results of the resolved analysis

Table 5 shows the predicted number of background events in the signal region, the number of events observed in the data, and the predicted yield for two potential signals. The numbers of predicted background events and observed events are in excellent agreement.

**Table 5:** The number of predicted background events in the *hh* signal region for the resolved analysis, compared to the data. Uncertainties correspond to the total uncertainties in the predicted event yields. The yield for two potential signals, SM non-resonant Higgs boson pair production and a 500 GeV $G^{*}_{\mathrm {KK}}$ in the bulk RS model with $k/\bar{M}_{\mathrm {Pl}}$ $=$ 1 are shown, with the uncertainties taken from Table 4

Sample	Signal region yield
Multijet	81.4 $\pm$ 4.9
$t\bar{t}$	5.2 $\pm$ 2.6
*Z*+jets	0.4 $\pm$ 0.2
Total	87.0 $\pm$ 5.6
Data	87
SM *hh*	0.34 $\pm$ 0.05
$G^{*}_{\mathrm {KK}}$ $(500\,{\mathrm {\ GeV}}{})$, $k/\bar{M}_{\mathrm {Pl}}$ $=$ 1	27 $\pm$ 5.9

**Fig. 6:**
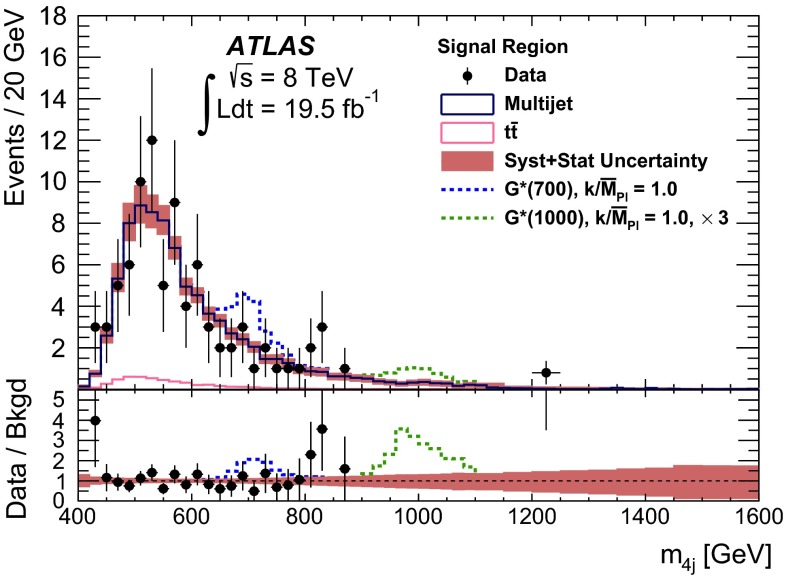
Distribution of the four-jet mass, $m_{4j}$, in the signal region of the resolved analysis for data (*points*) compared to the predicted background (*solid histograms*). The *filled blocks* represent the combined statistical and systematic uncertainty in the total background estimate. Two simulated signal $m_{\mathrm {4j}}$ peaks for the bulk RS model with $k/\bar{M}_{\mathrm {Pl}}$ $=$ 1 are shown as *dashed lines*

Figure 6 shows a comparison of the predicted $m_{\mathrm {4j}}$  background distribution to that observed in the data. The predicted background agrees with the observed distributions, with no significant deviation.

## Boosted analysis

### Event reconstruction

The boosted analysis differs from the resolved analysis primarily by the use of large-radius jets designed to contain the decay products of a single $h \rightarrow b\bar{b}$ decay. Those large-radius jets, denoted by the subscript J in the remainder of this paper, are reconstructed from locally calibrated topological clusters of calorimeter cells [63] using the ${\mathrm {anti-}}k_{t}$ jet clustering algorithm with a radius parameter of $R = 1.0$. To minimize the impact of energy depositions due to pile-up and the underlying event, the jets are trimmed [22]. This trimming algorithm reconstructs subjets within the large-*R* jet using the $k_{\mathrm t}$ algorithm with radius parameter $R_{\mathrm {sub}} = 0.3$, then removes any subjet with $p_{\text {T}}$ less than 5 % of the large-*R* jet $p_{\text {T}}$. Further calibration of both the energy and mass scales is applied as a function of $p_{\text {T}}$ and $\eta$ as determined from simulation and in situ measurements [65].

A novel aspect of the boosted technique presented here is the use of track-jets [23] to identify the presence of *b*-quarks inside the large-*R* jet. Such track-jets are built solely from tracks with $p_{\text {T}}$ $>$ 0.5 GeV and $|\eta |$  $<$ 2.5, satisfying a set of hit and impact parameter criteria to make sure that those tracks are consistent with originating from the primary vertex, thereby reducing the effects of pile-up. Track jets are reconstructed using the ${\mathrm {anti-}}k_{t}$ algorithm with $R = 0.3$. Flavour-tagging of those track-jets proceeds in the same way as for the $R = 0.4$ calorimeter jets used in the resolved analysis described in the previous section, except for a slightly looser requirement on the output of the MV1 neural network for a track-jet to be *b*-tagged. This leads to *b*-jets being *b*-tagged with an efficiency of 74 %, with a charm-jet rejection factor of approximately 4 and a light-quark or gluon jet rejection factor of around 65, as determined in an MC sample of $t\bar{t}$ events. The *b*-tagging efficiency for track-jets in the MC simulation is adjusted based on studies of $t\bar{t}$ events in the data (Sect. 5.4).

### Selection

**Fig. 7:**
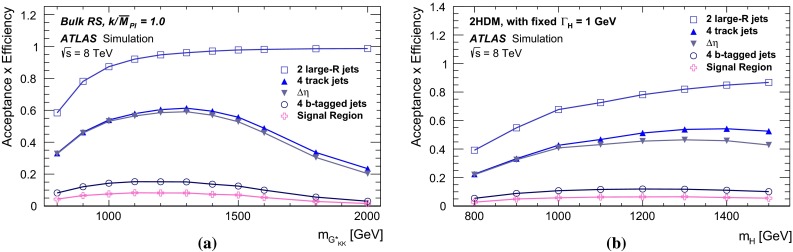
The selection efficiency as a function of resonance mass at each stage of the event selection for **a** $G^{*}_{\mathrm {KK}}$ $\rightarrow hh \rightarrow b\bar{b}b\bar{b}$ events and **b** $H \rightarrow hh \rightarrow b\bar{b}b\bar{b}$ events in the boosted analysis

The combined acceptance times efficiency at different stages of the event selection for the boosted analysis is shown in Fig. 7.

Events are required to contain at least two large-*R* jets with $p_{\text {T}} > 250$ GeV and $|\eta | < 2.0$. To suppress contamination from $t\bar{t}$ events, the leading jet is additionally required to have $p_{\text {T}} > 350$ GeV. This ensures that the top-quark decay products are typically fully contained in a single large-*R* jet with mass close to that of the top quark. These requirements are shown in Fig. 7 as “2 large-R jets”. Only the leading and subleading large-*R* jets are retained for further consideration.

Track jets are associated with large-*R* jets using “ghost association” [64, 72, 73]. Each of the leading and subleading large-*R* jets must have at least two track-jets ghost-associated with their respective untrimmed parents, where the track-jets must have $p_{\text {T}} > 20$ GeV and $|\eta | < 2.5$, as well as be consistent with originating from the primary vertex of the event (shown in Fig. 7 as “4 track-jets”). The drop in the $A \times \epsilon$ value at masses above 1500 GeV is due to the decrease in the angular separation between the two track-jets from the $h \rightarrow b\bar{b}$ decay to below $\Delta R = 0.3$.

To suppress contamination from multijet events, the two selected large-*R* jets in the event are required to have a separation $|\Delta \eta | < 1.7$. This requirement (shown in Fig. 7 as “$\Delta \eta$”) has only a small impact on the signal acceptance since high-mass resonances tend to produce jets that are more central than those from multijet background processes.

Selection of $h \rightarrow b\bar{b}$ candidates proceeds by requiring that both the leading and subleading track-jets associated with each of the two large-*R* jets satisfy the *b*-tagging selection (shown in Fig. 7 as “4 b-tagged jets”).

A final correction to the large-*R* jet four-momentum is applied to account for semileptonic *b*-hadron decays. If a muon passing the requirements outlined in Sect. 4.1 is ghost-associated with any of the selected *b*-tagged track-jets, its four-momentum is added to that of the large-*R* jet. If more than one muon is associated with a given track-jet, the muon closest to the track-jet axis is used. This correction improves the mass resolution for large-*R* jets in signal MC simulation, especially for the subleading jet.

The last requirement used to select signal event candidates is to require that the large-*R* jet mass is consistent with the Higgs boson mass. This requirement is defined identically to that for the resolved analysis in Eq. (1), except for the replacement of the small-*R* dijet mass with the large-*R* jet mass. The signal region is defined by the requirement $X_{hh} < 1.6$. This final selection is shown in Fig. 7 as “Signal Region”.

### Background estimation

After the 4-tag selection described in Sect. 5.2, the background composition is similar to that of the resolved analysis. Multijet events comprise approximately 90 % of the total background and are modelled entirely using data. The remaining $$\sim $$$$10~\%$$ of the background is $$t\bar{t}$$ events. The $$t\bar{t}$$ yield is determined using data, while the $$m_{\mathrm {2J}}$$ shape is taken from MC simulation. The $$Z+$$jets contribution is $$<$$1 % of the total background and is modelled using MC simulation. The background from all other sources – including processes featuring Higgs bosons – is negligible.

Estimation and validation of the background described below relies on two data samples defined as follows.

- The “4-tag” sample corresponds to the set of events that satisfy all the requirements detailed in Sect. 5.2, except that the final requirement on the mass of the leading and subleading large-*R* jets is not applied.
- The “2+3-tag” sample is identical to the 4-tag sample except for having only two or three of the four track-jets *b*-tagged. For events with only two *b*-tagged track-jets, both are required to be associated with the same large-*R* jet.

**Fig. 8:**
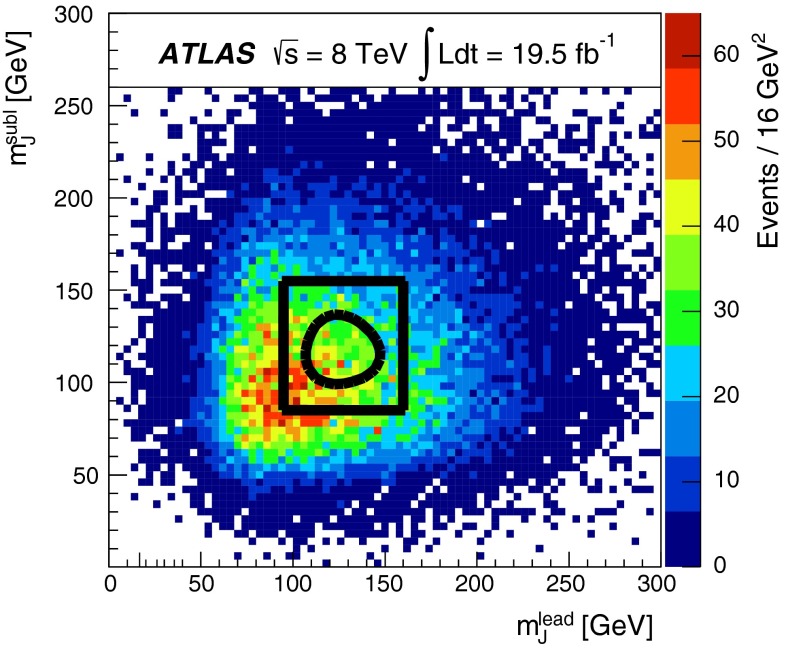
The leading–subleading large-*R* jet mass distribution for the 2-tag and 3-tag data sample in the boosted analysis. The signal region is the area surrounded by the *inner black contour line*, centred on $m_{\mathrm J}^{\mathrm {lead}} = 124$ GeV and $m_{\mathrm J}^{\mathrm {subl}} = 115$ GeV. The control region is the area inside the *outer black contour line*, excluding the signal region. The sideband region is the area outside the *outer contour line*

Both samples are further subdivided based on the large-*R* jet masses, with each subsample having a sideband region to determine the multijet background kinematics and a control region to validate the background estimate. The control region is defined by requirements on the mass of the leading and subleading large-*R* jets of $95 < m_{\mathrm J}^{\mathrm {lead}} < 160$ GeV and $85 < m_{\mathrm J}^{\mathrm {subl}} < 155$ GeV respectively, while excluding the signal region defined by $X_{hh} < 1.6$. The sideband region is complementary to the signal and control regions. Figure 8 illustrates the sideband and control regions with data from the $2+3$-tag sample.

The choice of control region (and consequently sideband region) ensures that the multijet background can be estimated by extrapolation of event yields and kinematic properties from the $2+3$-tag sample to the 4-tag sample with a normalization given by the relative event yields in the sideband region. Furthermore, the control region is chosen such that event kinematics in that region are representative of the kinematics in the signal region.

**Fig. 9:**
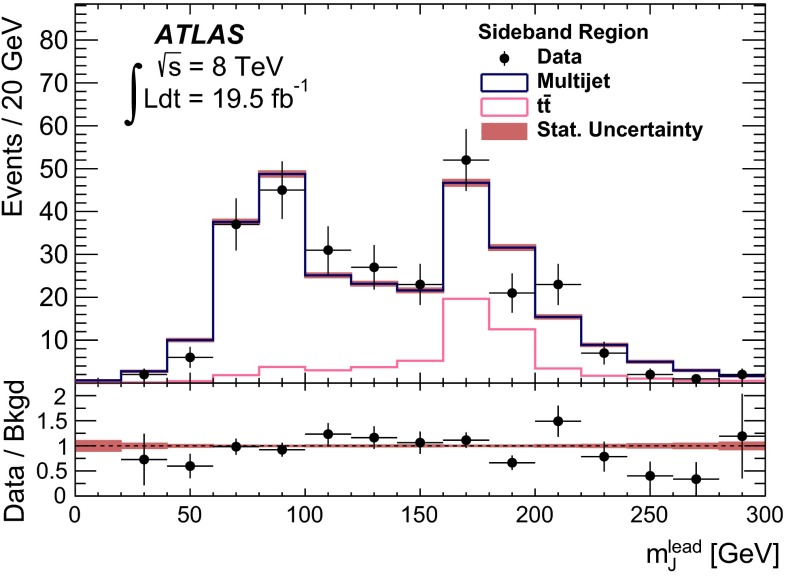
Leading large-*R* jet mass distribution for 4-tag events in the sideband region for data (*points*) and the two dominant sources of background for the boosted analysis. The normalization for each of those two background components is obtained with a fit to the data as described in the text

The estimated background yield in the 4-tag sample, $N^{\mathrm {4-tag}}_{\mathrm {bkg}}$, is computed according to

4 $$\begin{aligned} N^{\mathrm {4-tag}}_{\mathrm {bkg}} = \mu _{\mathrm {QCD}}\, N^{\mathrm {2+3-tag}}_{\mathrm {QCD}} + \alpha _{t\bar{t}}\, N^{\mathrm {4-tag}}_{t\bar{t}} + N^{\mathrm {4-tag}}_Z, \end{aligned}$$

where $N^{\mathrm {2+3-tag}}_{\mathrm {QCD}}$ is the number of multijet events in the $2+3$-tag data sample, $N^{\mathrm {4-tag}}_{t\bar{t}}$ and $N^{\mathrm {4-tag}}_Z$ are the numbers of events in the 4-tag $t\bar{t}$ and $Z+$jets MC samples. The parameter $\mu _{\mathrm {QCD}}$ corresponds to the ratio of multijet event yields in the 4-tag and $2+3$-tag data samples, as defined in Eq. (2), except for including both 2- and 3-tag events in the denominator. Finally, the parameter $\alpha _{t\bar{t}}$ is a scale factor designed to adjust the $t\bar{t}$ event yield from the MC simulation. Both $\mu _{\mathrm {QCD}}$ and $\alpha _{t\bar{t}}$ are extracted from a binned likelihood fit to the leading large-*R* jet mass distribution obtained in the sideband region of the 4-tag data sample, as depicted in Fig. 9. Due to the large minimum $p_{\text {T}}$ requirement for the leading large-*R* jet, much of the $t\bar{t}$ contribution is concentrated at high mass close to the top-quark mass. In this fit, the multijet distribution is extracted from the 2+3-tag data sample, after subtraction of the $t\bar{t}$ and $Z+$jets contributions predicted by the MC simulation. The $t\bar{t}$ and $Z+$jets distributions in the sideband region of the 4-tag data sample are taken from the MC simulation, but the $Z+$jets contribution is very small and its distribution is added to the multijet distribution for the fit. The resulting fit values are $\mu _{\mathrm {QCD}} = 0.0071 \pm 0.0007$ and $\alpha _{t\bar{t}} = 1.44 \pm 0.50$ with a correlation coefficient of $-0.67$ between these two parameters.

**Fig. 10:**
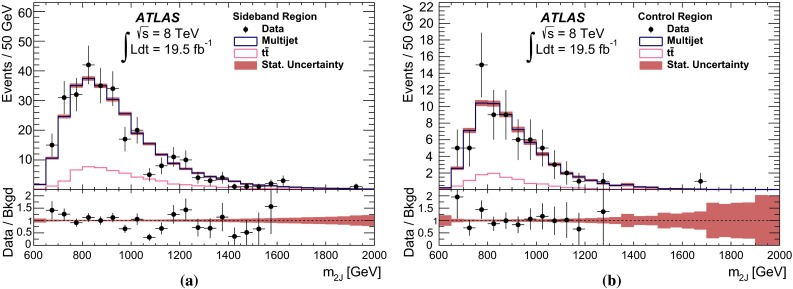
Dijet mass distributions for 4-tag events in the boosted analysis. **a** shows the sideband region and **b** the control region for data (*points*) and the expected background (*histograms*). The *filled blocks* represent the statistical uncertainty in the total background estimate

Figure 10a shows the dijet mass distribution for the 4-tag data sample in the sideband region with the background estimated using the above method. This figure indicates that the $2+3$-tag sample provides a valid description of the background kinematics in the 4-tag sample. The modelling of the background yield and kinematics is further validated by testing in the control region of the 4-tag data sample. Good agreement is observed between the data and the predicted background in various kinematical distributions for leading and subleading large-*R* jets, as well as for the dijet mass, as shown in Fig. 10b. The shapes of the $t\bar{t}$ kinematical distributions in the signal region are determined from the MC simulation requiring only three *b*-tagged track-jets instead of four due to the limited MC sample size. The number of $t\bar{t}$ events is then normalized to the expected yield in the 4-tag sample times $\alpha _{t\bar{t}}$. It was checked that this does not introduce a bias discernible with the statistical precision of the $t\bar{t}$ MC sample.

### Systematic uncertainties

Systematic uncertainties can be grouped into two classes: those affecting modelling of the signal as extracted from simulation and those arising from the background estimate.

The signal modelling is affected by two main sources of experimental uncertainty. One is related to large-*R* jets and the other is related to the efficiency for *b*-tagging track-jets. For large-*R* jets, the following uncertainties are accounted for: jet energy scale and resolution, as well as jet mass scale (JMS) and resolution (JMR). In the kinematic region relevant to this analysis, the JES uncertainty is below 2 % and that for JMS is $\sim$2–5 %. The JES uncertainty is derived with the $\gamma$-jet balance method for $p_{\text {T}} < 800$ GeV and the track-jets double-ratio method for $p_{\text {T}} > 800$ GeV, as described in Ref. [72]. The latter method is also used for the derivation of JMS uncertainties in the full $p_{\text {T}}$ range. An uncertainty of 20 % is applied to account for modelling of the jet energy and mass resolutions. The magnitude of this resolution uncertainty is estimated from studies of boosted *W* boson production performed using the 2012 data. Jet energy and mass uncertainties are treated as uncorrelated in the statistical analysis. The uncertainty in modelling the *b*-tagging efficiency for the track-jets used in this analysis is applied to the signal and $Z+$jets MC samples. It is extracted as a function of $p_{\text {T}}$ using the tag-and-probe method on a sample of dilepton events from semileptonic $t\bar{t}$ decays. The resulting uncertainty varies between 2 % and 7 %, with the largest value obtained for track-jets with $p_{\text {T}} > 100$ GeV. This uncertainty includes the following effects: statistical precision of the calibration data sample, choice of event generator and parton shower for the simulated $t\bar{t}$ sample, initial- and final-state radiation, and flavour composition. For $p_{\text {T}} > 250\,{\mathrm {\ GeV}}{}$, the uncertainties must be evaluated using MC simulation due to the small number of data events. Consequently, the uncertainties increase, reaching 14 % for $p_{\text {T}} > 600\,{\mathrm {\ GeV}}{}$. Studies in a $t\bar{t}$ data sample with a single-lepton$+$jets final state indicate that the presence of nearby jets does not have a measurable effect on the *b*-tagging efficiency and thus no additional uncertainty is required for nearby jets.

In addition to purely statistical sources of uncertainty, the background estimate is sensitive to the following other sources. The multijet background normalization is validated with the observed yield in the control region and the statistical uncertainty of this test is included as a systematic uncertainty. The shape of the $t\bar{t}$ background used in the fit shown in Fig. 9 is varied by extracting the shape from MC samples with zero, one, two or three *b*-tagged track-jets. Similarly, the uncertainty in the shape of other $t\bar{t}$ kinematical distributions is extracted from those samples. The uncertainty in the shape of the multijet background extracted from the sideband region of the $2+3$-tag sample is constrained by the level of agreement between the background prediction and the observed data in the control region following the procedure described in Sect. 4.4. Good agreement is observed between the data and the predicted background in both the sideband and control regions of the 4-tag sample as shown in Table 6.

**Table 6:** The number of events in data and predicted background events in the sideband and control regions of the 4-tag sample for the boosted analysis. The uncertainties are purely statistical

Sample	Sideband region	Control region
Multijet	221 $\pm$ 1	53.8 $\pm$ 0.6
$t\bar{t}$	52.8 $\pm$ 0.6	9.8 $\pm$ 0.3
$Z+$jets	3.80 $\pm$ 0.26	1.57 $\pm$ 0.17
Total	278 $\pm$ 1	65.2 $\pm$ 0.7
Data	281	68

**Table 7:** Summary of systematic uncertainties (expressed in percent) in the total background and signal event yields in the signal region for the boosted analysis. Uncertainties are provided for a resonance mass of 1.5 TeV in the context of the bulk RS model with $k/\bar{M}_{\mathrm {Pl}}$ $=$ 1 or 2, as well as for a Type-II 2HDM with $\Gamma _H = 1$ GeV, $\cos \left( \beta -\alpha \right) = -0.2$ and $\tan \beta = 1$

Source	Bkgd	$G^{*}_{\mathrm {KK}}$		*H*
		$$k/\bar{M}_{\mathrm {Pl}}$$ $$= $$1	$$k/\bar{M}_{\mathrm {Pl}}$$ $$=$$ 2	
Luminosity	0.2	2.8	2.8	2.8
JER	0.9	0.1	0.2	0.1
JES	0.4	0.1	2.5	0.1
JMR	4.3	13	13	12
JMS	1.3	18	17	16
*b*-tagging	–	21	20	21
Theoretical	–	2.0	2.0	2.0
Multijet	12	–	–	–
$t\bar{t}$	2.5	–	–	–
Bkgd stat	8.9	–	–	–
Total	15.9	33	28	30

Systematic uncertainties in both the background and signal event yields are summarized in Table 7. A 2.8 % luminosity uncertainty is applied to the $Z+$jets background and to the signal samples. The JER/JES/JMR/JMS uncertainties are applied to signal, $t\bar{t}$ and $Z+$jets samples. The track-jet *b*-tag uncertainty is applied only to the signal samples as the normalization and shape differences in the $t\bar{t}$ sample are accounted for through other sources of systematic uncertainty.

Theoretical uncertainties affecting the signal acceptance are also considered, as described in Sect. 4.4. These sources do not have significant dependence on the assumed resonance mass and the largest contribution is found to be due to the ISR modelling.

The uncertainty in the multijet event yield is derived from the difference between the predicted and observed multijet yields in the control region. This source of uncertainty is dominated by the statistical uncertainty in that region. The $t\bar{t}$ entry in Table 7 accounts for the shape uncertainty in the simulated $t\bar{t}$ leading-jet mass distribution in the sideband region used to fit for $\mu _{\mathrm {QCD}}$ and $\alpha _{t\bar{t}}$. This uncertainty is determined by comparing the shape of the 4-tag and 2-tag $t\bar{t}$ distributions. Finally, the “Bkgd stat” accounts for the statistical uncertainties in the extraction of $\mu _{\mathrm {QCD}}$ and $\alpha _{t\bar{t}}$. Uncertainties in the $m_{\mathrm {2J}}$ shape of the multijet and $t\bar{t}$ backgrounds are not listed in Table 7, as they do not affect the event yield, but are accounted for in the statistical analysis.

**Fig. 11:**
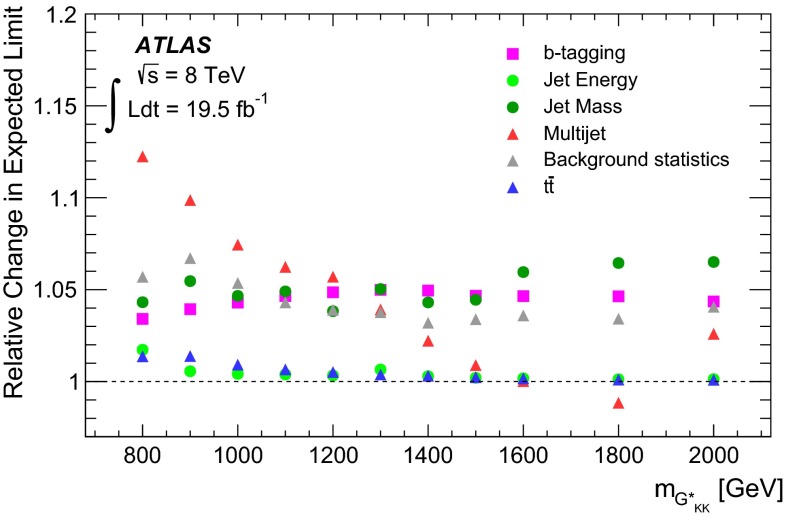
The individual relative impact of the systematic uncertainties considered in the boosted analysis on the expected $\sigma \left( pp \rightarrow G^{*}_{\mathrm {KK}} \rightarrow hh \rightarrow b\bar{b}b\bar{b}\right)$ 95 % confidence level exclusion limit, as a function of graviton mass. The calculation of the expected limit is described in Sect. 6. Only the mass-dependent uncertainties are shown. The impact is the ratio of the limit calculated using all systematic uncertainties sources to the limit calculated using all systematic uncertainty sources excluding those under investigation

Figure 11 presents the impact of each source of systematic uncertainty on the expected cross-section limit for the production of $$G^{*}_{\mathrm {KK}}$$ as a function of resonance mass with the choice $$k/\bar{M}_{\mathrm {Pl}}$$$$=$$ 1. These values are obtained following the statistical analysis described below while neglecting each source of uncertainty in turn. The multijet background uncertainty dominates for resonance masses below 1000 GeV, with *b*-tagging, large-*R* jet mass and the number of sideband data events for the background estimate becoming the most important at higher mass.

### Results of the boosted analysis

A total of 34 events is observed in the data whereas the background expectation is estimated to be $25.7 \pm 4.2$, see Table 8 for a breakdown of the various sources of background. The significance of this excess of events in the data is evaluated below.

**Table 8:** The number of predicted background events in the *hh* signal region, compared to the data for the boosted analysis. Errors correspond to the total uncertainties in the predicted event yields. The yield for a 1000 GeV $G^{*}_{\mathrm {KK}}$ in the bulk RS model with $k/\bar{M}_{\mathrm {Pl}}$ $=$ 1 is also given

Sample	Signal region yield
Multijet	23.5 $\pm$ 4.1
$t\bar{t}$	2.2 $\pm$ 0.9
$Z+$jets	0.14 $\pm$ 0.06
Total	25.7 $\pm$ 4.2
Data	34
$G^{*}_{\mathrm {KK}}$ $(1000\,{\mathrm {\ GeV}}{})$, $k/\bar{M}_{\mathrm {Pl}}$ $=$ 1	2.1 $\pm$ 0.6

**Fig. 12:**
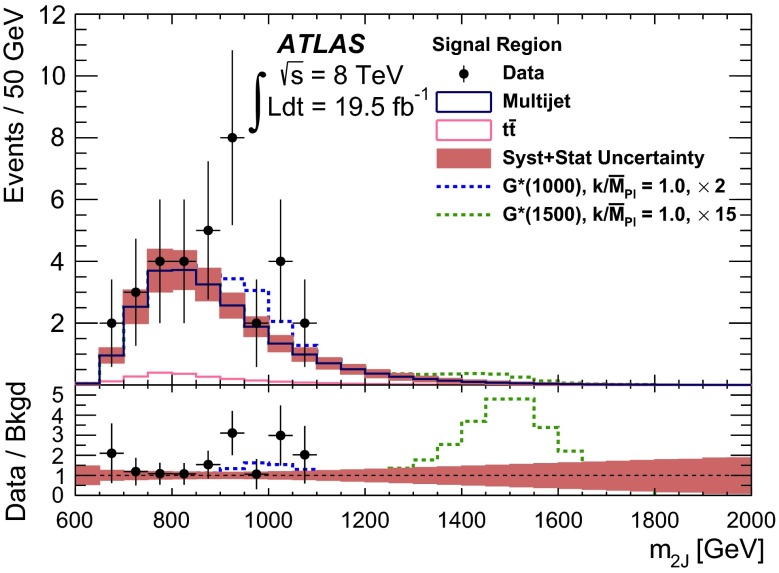
Dijet mass distributions for data (*points*) as well as expected background (*solid histograms*) in the signal region of the boosted analysis. The *filled blocks* represent the combined statistical and systematic uncertainty in the total background estimate. Two simulated $G^{*}_{\mathrm {KK}}$ signal peaks predicted by the bulk RS model with $k/\bar{M}_{\mathrm {Pl}}$ $=$ 1 are also shown as *dashed lines*

The dijet mass distribution in the signal region is shown in Fig. 12. For this distribution and the statistical analysis, the estimated background prediction from multijet ($t\bar{t}$) events is fit to an exponential function at masses above 900 (800) GeV and the associated uncertainty is propagated to the statistical analysis.

## Results

The results from the analyses in Sects. 4 and 5 are interpreted separately using the statistical procedure described in Ref. [1] and references therein. Hypothesized values of $\mu$, the global signal strength factor, are tested with a test statistic based on the profile likelihood ratio [74, 75]. In the profile likelihoods, the maximum likelihood values are obtained with the systematic uncertainties treated as independent, Gaussian or log-normal constraint terms. The statistical analysis described below is performed using data from the signal region solely. In the case of the search for non-resonant *hh* production, only the number of events passing the final selection is used whereas the $m_{\mathrm {4j}}$ or $m_{\mathrm {2J}}$ distributions are used in the case of the search for *hh* resonances.

### Background-only hypothesis tests

Tests of the background-only hypothesis ($\mu =0$) are carried out to determine if there are any statistically significant local excesses in the data. The significance of an excess is quantified using the local $p_{0}$, the probability that the background could produce a fluctuation greater than or equal to the excess observed in data. A global $p_0$ is also calculated for the most significant discrepancy, using background-only pseudo-experiments to derive a correction for the look-elsewhere effect across the mass range tested.

In the case of the resolved analysis, the largest deviation from the background-only hypothesis is found to be $$2.1\,\sigma $$ for a $$pp\rightarrow H \rightarrow hh \rightarrow b\bar{b}b\bar{b} $$ signal with fixed $$\Gamma _H = 1\,{\mathrm {\ GeV}}{}$$ at $$m_{\mathrm {4j}} = 1200$$ GeV. This corresponds to a global significance of $$0.42\,\sigma $$. The significance of any deviation for a $$G^{*}_{\mathrm {KK}}$$ signal with $$k/\bar{M}_{\mathrm {Pl}}$$$$=$$ 1 is very similar, albeit with slightly smaller local discrepancies as a result of the larger signal $$m_{\mathrm {4j}}$$ width.

In the case of the boosted analysis, the largest local deviation corresponds to the data excess at $$m_{\mathrm {2J}} ~\sim ~900$$ GeV apparent in Fig. 12, with a local significance of $$2.6\,\sigma $$ for $$pp\rightarrow G^{*}_{\mathrm {KK}} \rightarrow hh \rightarrow b\bar{b}b\bar{b} $$ with $$k/\bar{M}_{\mathrm {Pl}}$$$$=$$ 1. The global significance of this deviation corresponds to $$0.78\,\sigma $$.

Given these low significance values, the results of both analyses are consistent with the background-only hypothesis. Of the 117 events selected in the data by either the resolved or boosted analysis, only four events are common to both.

### Exclusion limits

The data are used to set upper limits on the cross-sections for the different benchmark signal processes. Exclusion limits are based on the value of the statistic $CL_{s}$  [76], with a value of $\mu$ regarded as excluded at 95 % confidence level (CL) when $CL_{s}$  is less than 5 %.

The non-resonant search is performed using the resolved analysis, since it has better sensitivity than the boosted analysis. Using the SM *hh* non-resonant production as the signal model, the observed 95 % CL upper limit is $\sigma (pp\rightarrow hh\rightarrow b\bar{b}b\bar{b}) = 202$ fb. This can be compared to the inclusive SM prediction (as defined in Sect. 3) of $\sigma (pp \rightarrow hh \rightarrow b\bar{b}b\bar{b}) = 3.6 \pm 0.5$ fb.

**Fig. 13:**
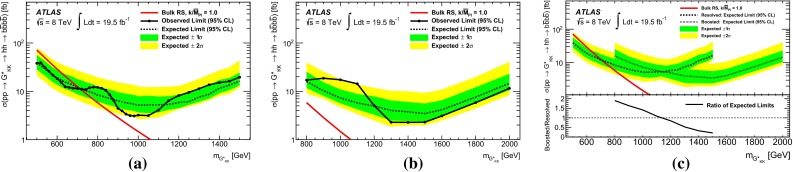
The expected and observed limits for the bulk RS model with $k/\bar{M}_{\mathrm {Pl}} =1$ for **a** the resolved analysis and **b** the boosted analysis. The overlay of expected limits is shown in **c**, demonstrating that the resolved analysis gives better sensitivity for $m_{G^{*}_{\mathrm {KK}}} < 1100\,{\mathrm {\ GeV}}{}$, while the boosted analysis is better for $m_{G^{*}_{\mathrm {KK}}} > 1100\,{\mathrm {\ GeV}}{}$. The *red curves* show the predicted cross-section as a function of resonance mass for the model considered

For the resonant Higgs boson pair production search, the resolved and boosted analyses offer their best sensitivity in complementary resonance mass regions. Figure 13 shows the expected and observed cross-section upper limits from each analysis for $pp\rightarrow G^{*}_{\mathrm {KK}} \rightarrow hh \rightarrow b\bar{b}b\bar{b}$ within the bulk RS model with $k/\bar{M}_{\mathrm {Pl}} =1$. The resolved analysis can be seen to give a more stringent expected exclusion limit for resonance masses up to 1100 GeV, while the boosted analysis offers better sensitivity beyond that mass. This motivates a simple combination of the separate exclusion limits from the resolved and boosted analyses. For each of the signal models, the limit for each mass point is taken from the analysis which offers the most stringent expected exclusion.

**Fig. 14:**
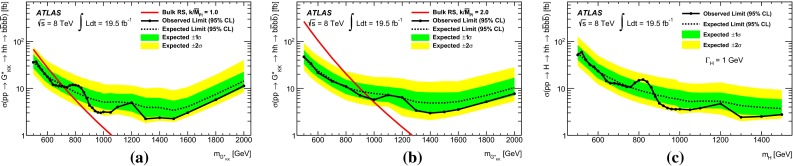
The combined expected and observed limit for $pp\rightarrow G^{*}_{\mathrm {KK}} \rightarrow hh \rightarrow b\bar{b}b\bar{b}$ in the bulk RS model with **a** $k/\bar{M}_{\mathrm {Pl}}$  $=$ 1 and **b** $k/\bar{M}_{\mathrm {Pl}}$ $=$ 2, as well as **c** $pp\rightarrow H \rightarrow hh \rightarrow b\bar{b}b\bar{b}$ with fixed $\Gamma _H = 1\,{\mathrm {\ GeV}}{}$. The *red curves* show the predicted cross-sections as a function of resonance mass for the models considered

Figure 14 shows the combined 95 % CL upper limits for three signal models: $$pp\rightarrow G^{*}_{\mathrm {KK}} \rightarrow hh \rightarrow b\bar{b}b\bar{b} $$ within the bulk RS model with $$k/\bar{M}_{\mathrm {Pl}}$$$$=$$ 1 and 2, and the $$pp\rightarrow H \rightarrow hh \rightarrow b\bar{b}b\bar{b} $$ with a fixed $$\Gamma _H = 1\,{\mathrm {\ GeV}}{}$$. The most stringent limits of $$\sigma \left( pp \rightarrow X \rightarrow hh \rightarrow b\bar{b}b\bar{b}\right) \sim 3\,$$fb are set in the range $$900 < m_X < 1600\,{\mathrm {\ GeV}}{}$$, where there is little expected background and either the resolved or boosted analysis provides good signal acceptance. The excluded mass ranges for the bulk RS KK graviton are shown in Table 9.

**Table 9:** Range of KK graviton masses excluded at 95 % confidence level for $k/\bar{M}_{\mathrm {Pl}} = 1.0, 1.5$ and 2.0

$k/\bar{M}_{\mathrm {Pl}}$	95 % CL excluded $G^{*}_{\mathrm {KK}}$ mass range [GeV]
1.0	500–720
1.5	500–800 and 870–910
2.0	500–990

The excluded mass range for the 2HDM is parameter dependent, principally because the production cross-section varies, but also because the exclusion limit depends on the parameter-dependent *H* boson width, $$\Gamma _H$$. The theoretical cross-section used to determine the 95 % CL excluded regions is the sum of the cross-sections of gluon-fusion production, vector-boson-fusion production and $b$-associated production.

**Fig. 15:**
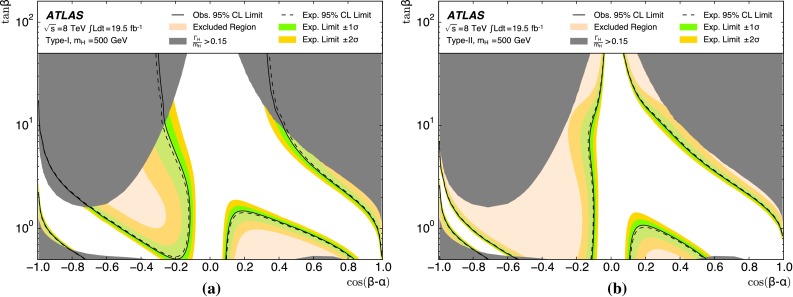
Excluded regions of the $(\cos (\beta - \alpha ), \tan \beta )$ parameter space for **a** the Type-I 2HDM signal model and **b** the Type-II 2HDM signal model. The *grey areas* demarcate the phase-space regions where $\Gamma _H/m_H > 0.15$, for which the cross-section limits have not been demonstrated to be reliable

**Fig. 16:**
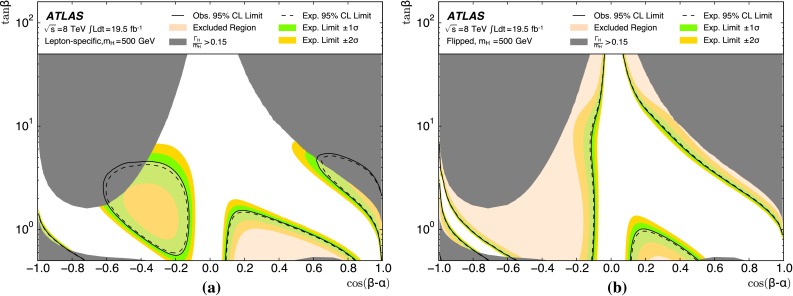
Excluded regions of the $(\cos (\beta - \alpha ), \tan \beta )$ parameter space for **a** the Lepton-specific 2HDM signal model and **b** the Flipped 2HDM signal model. The *grey areas* demarcate the phase-space regions where $\Gamma _H/m_H > 0.15$, for which the cross-section limits have not been demonstrated to be reliable

**Fig. 17:**
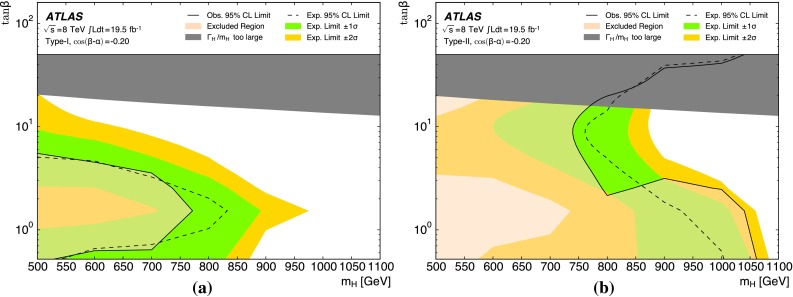
Excluded regions of the $(m_H, \tan \beta )$ parameter space for **a** the Type-I 2HDM signal model and **b** the Type-II 2HDM signal model. The *grey areas* demarcate the phase-space regions where $\Gamma _H/m_H$ is large ($\Gamma _H/m_H > 0.15$ for $m_H = 500\,{\mathrm {\ GeV}}{}$, increasing to $\Gamma _H/m_H > 0.23$ for $m_H = 1100\,{\mathrm {\ GeV}}{}$) and the limits have not been demonstrated to be reliable

**Fig. 18:**
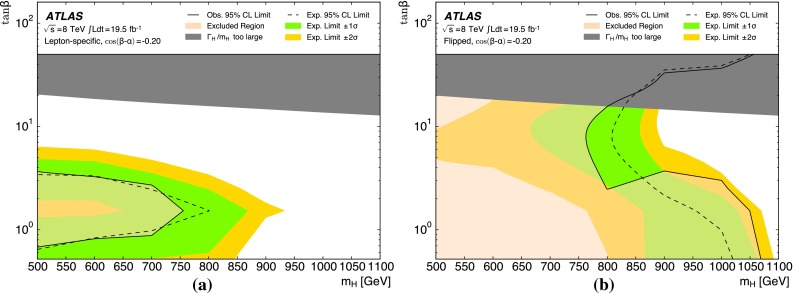
Excluded regions of the $(m_H, \tan \beta$ parameter space for **a** the Lepton-specific 2HDM signal model and **b** the Flipped 2HDM signal model. The *grey areas* demarcate the phase-space regions where $\Gamma _H/m_H$ is large ($\Gamma _H/m_H > 0.15$ for $m_H = 500\,{\mathrm {\ GeV}}{}$, increasing to $\Gamma _H/m_H > 0.23$ for $m_H = 1100\,{\mathrm {\ GeV}}{}$) and the limits have not been demonstrated to be reliable

The effects of $$\Gamma _H$$ are accounted for by creating $$m_H$$ distributions with a range of widths, $$0 < \Gamma _H/m_H \le 0.5$$, for each $$m_H$$ considered. These distributions are based on parameterizations which include resolution and acceptance effects combined with a Breit–Wigner line-shape. A grid of limits are calculated with each of these mass distributions. Then, for each point in $$m_H$$, $$\cos \left( \beta -\alpha \right) $$, and $$\tan \beta $$ space, the cross-section limit is determined by interpolating between the appropriate limits, based on the $$\Gamma _H$$ given by the model for that point. For the widest signals considered, the exclusion limits worsen by up to a factor of three. The exclusion regions determined through this process are shown as a function of $$\cos \left( \beta -\alpha \right) $$ and $$\tan \beta $$ for $$m_H=500\,{\mathrm {\ GeV}}{}$$ in Figs. 15 and 16, and as a function of $$m_H$$ and $$\tan \beta $$ for $$\cos \left( \beta -\alpha \right) = -0.2$$ in Figs. 17 and 18. The validity of the process has been tested using the widest available signals, gravitons in the bulk RS model with $$k/\bar{M}_{\mathrm {Pl}}$$$$=$$ 2. Phase-space regions with $$\Gamma _H$$ greater than these graviton widths are considered unvalidated and are shown in the figures as grey areas.

## Conclusions

Two searches for Higgs boson pair production with the ATLAS detector at the LHC using the $b\bar{b}b\bar{b}$ final state have been presented: one reconstructs Higgs boson candidates from pairs of nearby ${\mathrm {anti-}}k_{t}$*b*-tagged jets with $R = 0.4$; the other reconstructs Higgs boson candidates using trimmed ${\mathrm {anti-}}k_{t}$ jets with $R = 1.0$ matched to two *b*-tagged ${\mathrm {anti-}}k_{t}$ track-jets with $R = 0.3$. Thanks to the high expected $h \rightarrow b\bar{b}$ branching ratio and the large background rejection factors offered by the boosted dijet topology, the sensitivity for Higgs boson pair production is high, with a mass reach spanning the range between 500 and 2000 GeV. There is no evidence for any signal in 19.5 fb$^{-1}$of *pp* collision data with $\sqrt{s} = 8$ TeV. The largest deviation from the background-only hypothesis has a global significance of only 0.78 $\sigma$. The observed 95 % CL upper limit on $\sigma \left( pp \rightarrow X \rightarrow hh \rightarrow b\bar{b}b\bar{b}\right)$ is 3.2 (2.3) fb for narrow resonances with a mass of 1.0 (1.5) TeV.

Constraints are placed on several benchmark models. For the bulk RS model with $$k/\bar{M}_{\mathrm {Pl}} =1$$, KK gravitons in the mass range $$500\le $$$$m_{G^{*}_{\mathrm {KK}}}$$$$\le 720$$ GeV are excluded at the 95 % CL. For non-resonant signals, using Standard Model *hh* non-resonant production as the benchmark, the observed 95 % CL upper limit on $$\sigma (pp\rightarrow hh\rightarrow b\bar{b}b\bar{b})$$ is 202 fb, in good agreement with the expected exclusion. This is to be compared to a SM prediction of $$3.6 \pm 0.5$$ fb.

## Footnotes

1 ATLAS uses a right-handed coordinate system with its origin at the nominal interaction point (IP) in the centre of the detector and the *z*-axis along the beam pipe. The *x*-axis points from the IP to the centre of the LHC ring, and the *y*-axis points upwards. Cylindrical coordinates $(r, \phi )$ are used in the transverse plane, $\phi$ is the azimuthal angle around the beam pipe. The pseudorapidity, $\eta$, is defined in terms of the polar angle $\theta$ as $\eta = -\ln [\tan (\theta /2)]$.

2 Proton–proton collision vertices are reconstructed requiring that at least five tracks with $p_{\text {T}}$  $>$ 0.4 GeV are associated with a given vertex. The primary vertex is defined as the vertex with the highest summed track $p_{\text {T}} ^2$.

3 A jet is labelled as a *b*-jet if a *b*-quark with transverse momentum above 5 GeV exists within a cone of $\Delta R =0.3$ around the jet axis.

## References

[1] ATLAS Collaboration, Observation of a new particle in the search for the Standard Model Higgs boson with the ATLAS detector at the LHC. Phys. Lett. B **716**, 1 (2012). arXiv:1207.7214

[2] C.M.S. Collaboration, Observation of a new boson at a mass of 125 GeV with the CMS experiment at the LHC. Phys. Lett. B **716**, 30 (2012). arXiv:1207.7235

[3] ATLAS Collaboration, Measurements of Higgs boson production and couplings in diboson final states with the ATLAS detector at the LHC, Phys. Lett. B **726**, 88 (2013). arXiv:1307.1427

[4] C.M.S. Collaboration, Measurement of the properties of a Higgs boson in the four-lepton final state. Phys. Rev. D **89**, 092007 (2014). arXiv:1312.5353

[5] S. Dawson, S. Dittmaier, M. Spira, Neutral Higgs-boson pair production at hadron colliders: QCD corrections. Phys. Rev. D **58**, 115012 (1998). arXiv:hep-ph/9703387

[6] J. Grigo, J. Hoff, K. Melnikov, M. Steinhauser, On the Higgs boson pair production at the LHC. Nucl. Phys. B **875**, 1 (2013). arXiv:1305.7340

[7] de Florian D, Mazzitelli J (2013). Higgs Boson pair production at next-to-next-to-leading order in QCD. Phys. Rev. Lett..

[8] Agashe K, Davoudiasl H, Perez G, Soni A (2007). Warped gravitons at the LHC and beyond. Phys. Rev. D.

[9] Fitzpatrick L, Kaplan J, Randall L, Wang L-T (2007). Searching for the Kaluza–Klein graviton in bulk RS models. JHEP.

[10] Branco G (2012). Theory and phenomenology of two-Higgs-doublet models. Phys. Rep..

[11] Kribs GD, Martin A (2012). Enhanced di-Higgs production through light colored scalars. Phys. Rev. D.

[12] Grober R, Muhlleitner M (2011). Composite Higgs Boson pair production at the LHC. JHEP.

[13] Contino R (2012). Anomalous couplings in double Higgs production. JHEP.

[14] ATLAS Collaboration,Search for Higgs Boson pair production in the $\gamma\gamma b\bar{b}$ Final State using $pp$ Collision Data at $\sqrt{s} = 8$ TeV from the ATLAS Detector Phys. Rev. Lett. **114**, 081802 (2015). arXiv:1406.5053 10.1103/PhysRevLett.114.081802 25768755

[15] Collaboration CMS (2014). Searches for heavy Higgs bosons in two-Higgs-doublet models and for $$t\rightarrow ch$$ decay using multilepton and diphoton final states in $$pp$$ collisions at 8 TeV. Phys. Rev. D.

[16] CMS Collaboration, Search for resonant pair production of Higgs bosons decaying to two bottom quark–antiquark pairs in proton–proton collisions at 8 TeV. arXiv:1503.04114

[17] Cooper B, Konstantinidis N, Lambourne L, Wardrope D (2013). Boosted $$hh\rightarrow b\overline{b}b\overline{b}$$: a new topology in searches for TeV-scale resonances at the LHC. Phys. Rev. D.

[18] Gouzevitch M (2013). Scale-invariant resonance tagging in multijet events and new physics in Higgs pair production. JHEP.

[19] de Lima D, Papaefstathiou A, Spannowsky M (2014). Standard model Higgs boson pair production in the $$\left(b\bar{b}\right)\left(b\bar{b}\right)$$ final state. JHEP.

[20] Cacciari M, Salam GP, Soyez G (2008). The anti-k$$_t$$ jet clustering algorithm. JHEP.

[21] ATLAS Collaboration, Calibration of the performance of $$b$$ and light-flavour jets in the 2012 ATLAS data. http://cds.cern.ch/record/1741020

[22] Krohn D, Thaler J, Wang L-T (2010). Jet trimming. JHEP.

[23] ATLAS Collaboration, Flavor tagging with track jets in boosted topologies with the ATLAS Detector. Technical Report ATL-PHYS-PUB-2014-013 (2014). http://cds.cern.ch/record/1712269

[24] ATLAS Collaboration, The ATLAS experiment at the CERN Large Hadron Collider. JINST **3**, S08003 (2008)

[25] ATLAS Collaboration, Performance of the ATLAS trigger system in 2010 (2010). Eur. Phys. J. C.

[26] ATLAS Collaboration, Improved luminosity determination in pp collisions at $$\sqrt{s}=7$$ TeV using the ATLAS detector at the LHC. Eur. Phys. J. C **73**, 2518 (2013). arXiv:1302.4393 10.1140/epjc/s10052-013-2518-3 PMC4370906 25814867

[27] Alwall J (2011). MadGraph 5: going beyond. JHEP.

[28] O. Antipin, T. Hapola, Tools for Discovering Extra Dimensions (2013). http://cp3-origins.dk/research/units/ed-tools

[29] Sjostrand T, Mrenna S, Skands PZ, Brief A (2008). Introduction to PYTHIA 8.1. Comput. Phys. Commun..

[30] Stump D (2003). Inclusive jet production, parton distributions, and the search for new physics. JHEP.

[31] Harlander RV, Liebler S, Mantler H (2013). SusHi: a program for the calculation of Higgs production in gluon fusion and bottom-quark annihilation in the Standard Model and the MSSM. Comput. Phys. Commun..

[32] Harlander R, Kant P (2005). Higgs production and decay: analytic results at next-to-leading order QCD. JHEP.

[33] Harlander RV, Kilgore WB (2003). Higgs boson production in bottom quark fusion at next-to-next-to leading order. Phys. Rev. D.

[34] Harlander RV, Kilgore WB (2002). Next-to-next-to-leading order Higgs production at hadron colliders. Phys. Rev. Lett..

[35] Aglietti U, Bonciani R, Degrassi G, Vicini A (2004). Two loop light fermion contribution to Higgs production and decays. Phys. Lett. B.

[36] Bonciani R, Degrassi G, Vicini A (2011). On the generalized harmonic polylogarithms of one complex variable. Comput. Phys. Commun..

[37] R. Harlander, M. Kramer, M. Schumacher, Bottom-quark associated Higgs-boson production: reconciling the four- and five-flavour scheme approach. arXiv:1112.3478

[38] Dawson S, Jackson C, Reina L, Wackeroth D (2004). Exclusive Higgs boson production with bottom quarks at hadron colliders. Phys. Rev. D.

[39] Dittmaier S, Kramer M, Spira M (2004). Higgs radiation off bottom quarks at the Tevatron and the CERN LHC. Phys. Rev. D.

[40] LHC Higgs Cross Section Working Group Collaboration, S. Heinemeyer et al., *Handbook of LHC Higgs Cross Sections: 3. Higgs Properties*. arXiv:1307.1347

[41] Eriksson D, Rathsman J, Stal O (2010). 2HDMC: two-Higgs-doublet model calculator physics and manual. Comput. Phys. Commun..

[42] Plehn T, Spira M, Zerwas P (1996). Pair production of neutral Higgs particles in gluon–gluon collisions. Nucl. Phys. B.

[43] Frederix R (2014). Higgs pair production at the LHC with NLO and parton-shower effects. Phys. Lett. B.

[44] Nason P (2004). A new method for combining NLO QCD with shower Monte Carlo algorithms. JHEP.

[45] Frixione S, Nason P, Oleari C (2007). Matching NLO QCD computations with Parton Shower simulations: the POWHEG method. JHEP.

[46] Frixione S, Nason P, Ridolfi G (2007). A Positive-weight next-to-leading-order Monte Carlo for heavy flavour hadroproduction. JHEP.

[47] Alioli S, Nason P, Oleari C, Re E (2010). A general framework for implementing NLO calculations in shower Monte Carlo programs: the POWHEG BOX. JHEP.

[48] T. Sjostrand, S. Mrenna, P.Z. Skands, PYTHIA 6.4 physics and manual. JHEP **05**, 026 (2006). arXiv:hep-ph/0603175

[49] Lai H-L (2010). New parton distributions for collider physics. Phys. Rev. D.

[50] Cacciari M (2012). Top-pair production at hadron colliders with next-to-next-to-leading logarithmic soft-gluon resummation. Phys. Lett. B.

[51] Bärnreuther P, Czakon M, Mitov A (2012). Percent level precision physics at the Tevatron: first genuine NNLO QCD corrections to $$q \bar{q} \rightarrow t \bar{t} + X$$. Phys. Rev. Lett..

[52] Czakon M, Mitov A (2012). NNLO corrections to top-pair production at hadron colliders: the all-fermionic scattering channels. JHEP.

[53] Czakon M, Mitov A (2013). NNLO corrections to top pair production at hadron colliders: the quark-gluon reaction. JHEP.

[54] Czakon M, Fiedler P, Mitov A (2013). The total top quark pair production cross-section at hadron colliders through O($$\alpha _{S}^{4}$$). Phys. Rev. Lett..

[55] Czakon M, Mitov A (2014). Top++: a program for the calculation of the top-pair cross-section at hadron colliders. Comput. Phys. Commun..

[56] T. Gleisberg et al., Event generation with SHERPA 1.1. JHEP **02**, 007 (2009). arXiv:0811.4622

[57] Alioli S, Nason P, Oleari C, Re E (2011). Vector boson plus one jet production in POWHEG. JHEP.

[58] ATLAS Collaboration, Measurement of the cross-section of high transverse momentum $Z\rightarrow b\bar{b}$ production in protonproton collisions at $\sqrt{s} = 8$ TeV with the ATLAS detector. Phys. Lett. B **738**, 25 (2014). arXiv:1404.7042

[59] GEANT4 Collaboration, S. Agostinelli et al., *GEANT4: a simulation toolkit*. Nucl. Instrum. Method A **506**, 250 (2003)

[60] ATLAS Collaboration, The ATLAS simulation infrastructure. Eur. Phys. J. C **70**, 823 (2010). arXiv:1005.4568

[61] ATLAS Collaboration, Summary of ATLAS Pythia 8 tunes. ATL-PHYS-PUB-2012-003. http://cdsweb.cern.ch/record/1474107

[62] B. Hespel, E. Vryonidou, *Higgs Pair Production* (2015). https://cp3.irmp.ucl.ac.be/projects/madgraph/wiki/HiggsPairProduction

[63] ATLAS Collaboration, Jet energy measurement with the ATLAS detector in proton–proton collisions at $$\sqrt{s}=7$$ TeV. Eur. Phys. J. C **73**, 2304 (2013). arXiv:1112.6426 10.1140/epjc/s10052-013-2306-0 PMC4371084 25814854

[64] Cacciari M, Salam GP, Soyez G (2008). The catchment area of jets. JHEP.

[65] ATLAS Collaboration, Jet energy measurement and its systematic uncertainty in proton–proton collisions at $$\sqrt{s}=7$$ TeV with the ATLAS detector. Eur. Phys. J. C **75**, 17 (2015). arXiv:1406.0076 10.1140/epjc/s10052-014-3190-y PMC4684939 26709345

[66] ATLAS Collaboration, Measurement of the muon reconstruction performance of the ATLAS detector using 2011 and 2012 LHC proton–proton collision data. Eur. Phys. J. C **74**, 3130 (2014). arXiv:1407.3935 10.1140/epjc/s10052-014-3130-x PMC4371046 25814875

[67] ATLAS Collaboration, Pile-up subtraction and suppression for jets in ATLAS. ATLAS-CONF-2013-083. http://cds.cern.ch/record/1570994

[68] ATLAS Collaboration, Calibration of $$b$$-tagging using dileptonic top pair events in a combinatorial likelihood approach with the ATLAS experiment. ATLAS-CONF-2014-004. http://cds.cern.ch/record/1664335

[69] Martin A, Stirling W, Thorne R, Watt G (2009). Parton distributions for the LHC. Eur. Phys. J. C.

[70] Ball RD (2013). Parton distributions with LHC data. Nucl. Phys. B.

[71] J. Wenninger, Energy calibration of the LHC beams at 4 TeV. Technical Report CERN-ATS-2013-040, CERN, Geneva (2013). http://cds.cern.ch/record/1546734

[72] ATLAS Collaboration, Performance of jet substructure techniques for large-$R$ jets in proton-proton collisions at $\sqrt{s} = 7$ TeV using the ATLAS detector. JHEP **09**, 076 (2013). arXiv:1306.4945

[73] Cacciari M, Salam GP (2008). Pileup subtraction using jet areas. Phys. Lett. B.

[74] Cowan G, Cranmer K, Gross E, Vitells O (2011). Asymptotic formulae for likelihood-based tests of new physics. Eur. Phys. J. C.

[75] Cowan G, Cranmer K, Gross E, Vitells O (2013). Erratum: Asymptotic formulae for likelihood-based tests of new physics. Eur. Phys. J. C.

[76] Read AL (2002). Presentation of search results: the CL(s) technique. J. Phys..

